# Zebrafish *hoxd4a* Acts Upstream of *meis1.1* to Direct Vasculogenesis, Angiogenesis and Hematopoiesis

**DOI:** 10.1371/journal.pone.0058857

**Published:** 2013-03-15

**Authors:** Aseervatham Anusha Amali, Lawrence Sie, Christoph Winkler, Mark Featherstone

**Affiliations:** 1 School of Biological Sciences, Nanyang Technological University, Singapore, Singapore; 2 Department of Biological Sciences, National University of Singapore, Singapore, Singapore; Center for Interdisciplinary Research in Biology (CIRB) is a novel Collège de France/CNRS/INSERM, France

## Abstract

Mice lacking the 4^th^-group paralog *Hoxd4* display malformations of the anterior vertebral column, but are viable and fertile. Here, we report that zebrafish embryos having decreased function of the orthologous *hoxd4a* gene manifest striking perturbations in vasculogenesis, angiogenesis and primitive and definitive hematopoiesis. These defects are preceded by reduced expression of the hemangioblast markers *scl1*, *lmo2* and *fli1* within the posterior lateral plate mesoderm (PLM) at 13 hours post fertilization (hpf). Epistasis analysis revealed that *hoxd4a* acts upstream of *meis1.1* but downstream of *cdx4* as early as the shield stage in ventral-most mesoderm fated to give rise to hemangioblasts, leading us to propose that loss of *hoxd4a* function disrupts hemangioblast specification. These findings place *hoxd4a* high in a genetic hierarchy directing hemangioblast formation downstream of *cdx1*/*cdx4* and upstream of *meis1.1*. An additional consequence of impaired *hoxd4a* and *meis1.1* expression is the deregulation of multiple Hox genes implicated in vasculogenesis and hematopoiesis which may further contribute to the defects described here. Our results add to evidence implicating key roles for Hox genes in their initial phase of expression early in gastrulation.

## Introduction

The processes of vasculogenesis, angiogenesis and hematopoiesis establish the circulatory system and blood lineages of the embryo [1]–[4]. In zebrafish, the precursors of the vascular and hematopoietic systems derive from unipotential progenitors as well as a common progenitor known as the hemangioblast [5]. The hemangioblast is identifiable by the expression of the *scl* gene within the PLM [1], [3]. The first blood vessels are laid down through the process of vasculogenesis whereby angioblasts under the influence of vascular endothelial growth factor (VEGF) migrate and then aggregate as endothelial cells to form a vascular cord [2]. Subsidiary blood vessels are then formed by sprouting from existing vessels in a process termed angiogenesis [2].

In zebrafish, markers of vasculogenesis are apparent at 12 hpf with the formation of *scl1*-expressing cells in the anterior lateral plate mesoderm (ALM) and *scl*- and *fli1*-expressing angioblasts in the PLM [2], [3]. Between 18 and 22 hpf, angioblasts migrate to the midline, merge and lumenize to form the primary blood vessel [2]. Recent evidence suggests that the second major axial vessel, the posterior cardinal vein (PCV) is produced from the ventral side of the primary vessel through a process of ventral sprouting between 21 and 24 hpf; it expresses *ephb4a* and is distinguished from the dorsal aorta (DA) which expresses the arterial marker *efnb2a* [2]. From 20 hpf, new vessels sprout from the preexisting DA and at 32 hpf from the PCV [2]. From the DA, the primary intersegmental vessels (ISV) sprout dorsally, followed by the secondary ISV from PCV [2].

As in other vertebrates, blood development in zebrafish initiates with primitive hematopoiesis and is then followed by definitive hematopoiesis which establishes the adult blood lineages [1], [3]. In mammals and birds, the extra-embryonic yolk sac is the primary site of hematopoiesis, while in zebrafish markers of primitive hematopoiesis are apparent at 10 to 11 hpf (2 somite stage) in the ALM and PLM as revealed by the expression of hemangioblast markers *scl1* and *lmo2* [1], [3]. The first signs of commitment to primitive hematopoiesis are given by the expression of *gata1* in a subset of *scl1* positive cells of the PLM [1]. The myeloid progenitors and some endothelial cells are produced in the ALM. By 14 hpf (10 somite stage), precursors in the PLM commence migration to the midline and there form the intermediate cell mass (ICM), the major site of primitive hematopoiesis in the posterior of the embryo [1], [3]. Circulation initiates at 24 hpf following which a transient site of erythromyelopoiesis, the posterior blood island (PBI), functions over the next 12 hours [4].

The second and definitive wave of hematopoiesis begins in the ventral wall of the DA at 24 hpf [1], [3]. The ventral DA is considered to be the counterpart of the aorta-gonad-mesonephros (AGM) region of amniotes. HSCs arise in the AGM beginning at 24 hpf and are identifiable by expression of *runx1* and *cmyb*. Starting at 48 hpf, the HSCs then seed what will become the hematopoietic stroma of the caudal hematopoietic tissue (CHT) at 3 dpf, followed by expansion and differentiation [4], [6]. The CHT, which assumes the role of the fetal liver in mammals, is responsible for definitive hematopoiesis until the kidney marrow becomes the final hematopoietic site beginning at 4 days post-fertilization (dpf). The kidney marrow and the thymus become the lifelong primary hematopoietic sites in larval and adult life [1], [3].

Several genes have been implicated in the control of successive steps in the formation of blood and the vasculature. In zebrafish, the earliest acting gene to be identified to date is *cloche* [7] which may be synonymous with the acyltransferase-encoding gene *lycat* [8]. Also very high in the genetic hierarchy governing vasculogenesis and hematopoiesis are *cdx1* and *cdx4* [9], [10]. The severe hematopoietic defects seen in zebrafish *cdx4* single mutants and *cdx1/cdx4* doubly deficient embryos are accompanied by the down-regulation of the hemangioblast and hematopoietic stem cell (HSC) marker *scl1* and a number of Hox genes, and normal hematopoiesis can be rescued by the forced expression of *hoxa9a* [10].

Hox genes have also been implicated in hematopoietic and vasculogenic processes in mammals [11]–[13]. Their protein products are characterized by a conserved DNA-binding homeodomain and bind DNA cooperatively with members of the TALE homeoprotein family of cofactors [14] such as MEIS (Myeloid Ecotropic Integration Site), PBX (Pre-B-Cell Leukemia Homeobox), and PREP/PKNOX (Pbx Knotted Homeobox). There are 39 Hox genes in human and mouse organized into four clusters [15]. Extensive evidence implicates both Hox gene products and their cofactors in hematopoietic function [14], [16]–[21]. The ablation of any of several murine Hox genes, including *Hoxb4*, leads to defects in blood lineages. *Hoxb4* and indeed all remaining Hox genes of paralog group 4 (*Hoxa4*, *Hoxc4*, *Hoxd4*) display potent HSC-promoting functions [13], [22]. Similar activities are shared by the murine HOX cofactors PREP1, MEIS1 and PBX1 as revealed by hematopoietic and cardiac deficiencies in corresponding mutant animals [14], [21], [23]–[27]. The natural role for *Hox* and *Meis1* genes in normal hematopoiesis is further reflected in their well-documented contributions to human and murine leukemias [13], [28]–[31].

In teleosts, a genome duplication event generated 8 *Hox* clusters, with zebrafish retaining 7 clusters and 47 genes following evolutionary loss [32]. In zebrafish, *hoxb6b*, *hoxb7a*, and *hoxa9a* are known to regulate primitive hematopoiesis and are required for hematopoietic stem cell formation [9], [10], while deficiencies in *pbx* and *meis* function strongly compromise primitive and definitive hematopoiesis as well as the vasculature [18]–[20] pointing to a conservation of function across vertebrates.

Mice lacking *Hoxd4* function show abnormalities of the anterior-most vertebrae, the atlas and axis, but are viable and fertile [33], [34]. We explored the extent to which function was conserved in zebrafish by assessing the phenotype of *hoxd4a* loss-of-function morphants. We demonstrate a surprising role for zebrafish *hoxd4a* in vasculogenesis, angiogenesis and primitive and definitive hematopoiesis. Defects resulting from *hoxd4a* deficiency can be rescued by capped mRNA for *hoxd4a*, *meis1.1*, *scl,* and *fli1* but not by *cdx4.* Impaired *meis1.1* function following *hoxd4a* knockdown leads to widespread *Hox* gene deregulation including the reduced expression of *Hox* genes previously implicated in hematopoiesis, vasculogenesis and angiogenesis. The *cdx4*, *hoxd4a* and *meis1.1* genes are spatially and temporally co-expressed in shield-stage embryos within the ventral-most presumptive mesoderm, the site from which fate mapping studies have shown the hemangioblast to arise [5], [35], [36]. Moreover, shield-stage *hoxd4a* morphants display decreased *meis1.1* expression in ventral-most presumptive mesoderm, and this *meis1.1* expression is rescued by prior injection of capped *hoxd4a* mRNA. Thus, our data indicate that *hoxd4a* functions at the earliest times and near the top of a regulatory hierarchy directing hemangioblast formation, acting downstream or in parallel to *cdx* genes, but upstream or parallel to *meis1.1*. Another consequence of impaired *hoxd4a* and *meis1.1* function is the subsequent deregulation of multiple Hox genes previously implicated in vasculogenic and hematopoietic processes. We conclude that *hoxd4a* has acquired (or retained) a much more important role in hematopoiesis and vasculogenesis than observed for its murine ortholog *Hoxd4*. Additionally, our results add to a growing body of evidence defining functions for Hox genes during their early phase of expression at gastrulation, well before they are deployed along the antero-posterior axis proper.

## Methods

### Ethics Statement

All animal work conformed to the Institutional Animal Care and Use Committee (IACUC) guidelines at Nanyang Technological University and was reviewed and approved under protocol number ARF SBS/NIE-A 0144 AZ.

### **Zebrafish**

Wild-type AB and transgenic (*fli1*:EGFP) [37] and Tg(gata1:dsRed) [38] lines of zebrafish were maintained as described [39]. Zebrafish embryos were staged as detailed previously [40].

### Antisense Morpholino and mRNA Microinjection

Antisense morpholino oligonucleotides (MOs) were obtained from Gene Tools Inc and injected into zebrafish embryos at 1–4 cell stage. Splice MOs targeting the 5′ splice site intron-exon junction (splice acceptor) are designated as follows MO1∶5′-GTT CAC TGT GAA GGA CAA AAT CAC A-3′ and exon-intron junction (splice donor), MO2∶5′- GCA AAG AGA GTG GAT CTT ACC CGT A-3′. MOs were diluted in Danieau’s buffer (0.4 mM MgSO_4_, 0.6 mM CaCl_2_, 0.7 mM KCl, 58 mM NaCl, and 5 mM Hepes, pH 7.6). Optimal doses for each MO were tested based on phenotypic effects, and each experiment was performed in parallel with a non-specific MO (standard control MO supplied by GeneTools Inc) injected at the same concentration.

Full-length cDNA for *hoxd4a* was generated by PCR using the primers shown in **Table S1** **in File S1** and cloned into pCR®II-TOPO®. The mMESSAGE mMACHINE Kit (Ambion) was used to synthesize capped mRNA. All mRNAs used for rescue experiments were assessed over a range of concentrations and the following amounts were chosen as the minimum sufficient to induce rescue: *scl1* (100 pg), *fli1a* (30 pg), *meis1.1* (100 pg) and *hoxd4a* (50 pg). All injections were done at the 1-cell stage.

### qRT-PCR

Total RNA was extracted from embryos at 26–28 hpf using the PureLink™ Micro-to-Midi™ Total RNA Purification System (Invitrogen). 1 µg total RNA was treated with 1 U DNase I (Fermentas) at 37°C for 15 min and used for reverse transcription with SuperScript® III First-Strand Synthesis (Invitrogen). Quantitative reverse transcriptase PCR (qRT-PCR) was performed using SYBR GreenER™ qPCR SuperMix (Invitrogen) on a BioRad iCycler iQ5. The data (in biological triplicates) were normalized against zebrafish β-actin. The sequences of the oligonucleotides used for qRT-PCR are given in **Table S2 in File S1**.

### Whole Mount *in situ* Hybridization and Imaging

Whole mount *in situ* hybridization (WISH) was performed as previously described [41]. For *nkx2.5*, a PCR fragment was amplified from 26–28 hpf embryonic cDNA using the primers shown in **Table S1 in File S1**. The reverse primer incorporated a T7 promoter allowing the PCR product to be used directly for probe production. DIG- labeled antisense RNA probes were transcribed from linearized template using T3, T7 or SP6 RNA polymerase (Roche). Probes for *hoxb6b* and *hoxb7a* were derived by RT-PCR-mediated amplification from RNA from 26–28 hpf embryos using primers described in Wan et al [42]. Embryos were incubated with anti-DIG antibody (Roche) and probes were detected using NBT/BCIP (nitro blue tetrazolium chloride/5-bromo-4-chloro-3-indolyl phosphate, toluidine salt) from Roche. Images were obtained on a Zeiss lumar V.12 stereo microscope with an Axio Cam MRc (Zeiss) and Axio Vision software. Some embryos were then dissected away from the yolk and flat-mounted prior to photography.

### Alkaline Phosphatase Staining of Blood Vessels

Embryos at 72 hpf were fixed with 4% paraformaldehyde in PBS (phosphate buffered saline) at room temperature for 30 min, followed by treatment with pre-cooled acetone for 30 min at −20°C. After rinsing with PBS twice (5 min each), the embryos were equilibrated with NTMT buffer (100 mM Tris pH 9.5, 50 mM MgCl_2_, 100 mM NaCl, 0.1% Tween 20) for three times (each for 15 min) at room temperature. For alkaline phosphatase staining, the embryos were incubated in NBT/BCIP (Roche) solution for 30 min.

### O-dianisidine Staining

Staining with o-dianisidine was carried out as described [43]. Control and MO-injected embryos at 48 and 72 hpf were manually dechorionated and fixed with 4% paraformaldehyde overnight. Fixed embryos were washed three times in PBS and then incubated in the staining buffer (0.6 mg/ml o-dianisidine, 10 mM sodium acetate (pH 5.2), 0.65% hydrogen peroxide, and 40% ethanol) for 15 min in the dark. Stained embryos were cleared and stored in benzyl benzoate/benzyl alcohol (2∶1, vol/vol).

### Flow Cytometry

Control- and morpholino-injected Tg(gata1:dsRed) embryos (150 embryos each) were dechorionated manually at 48 hpf, rinsed for 15 min in calcium-free Ringer’s solution and passed five times through a 200 µl pipette tip to remove the yolk. The embryos were dissociated in 0.25% trypsin and 1 mM EDTA for 60 minutes at 28.5°C, during which the sample was passed six times through a 200 µl pipette tip every 10 minutes in order to obtain a single cell suspension. The dissociated cell suspension was centrifuged at 1000 g for 9 min at 4°C, the supernatant discarded, and the cells resuspended in ice cold 0.9× PBS plus 5% fetal bovine serum and passed by gravity through a 40 µm nylon mesh filter. Flow cytometry was performed on a BD LSII instrument (BD Biosciences). We used wild type zebrafish embryos as a negative control.

## Results

### Expression Pattern of *hoxd4a*

**Figure 1:**
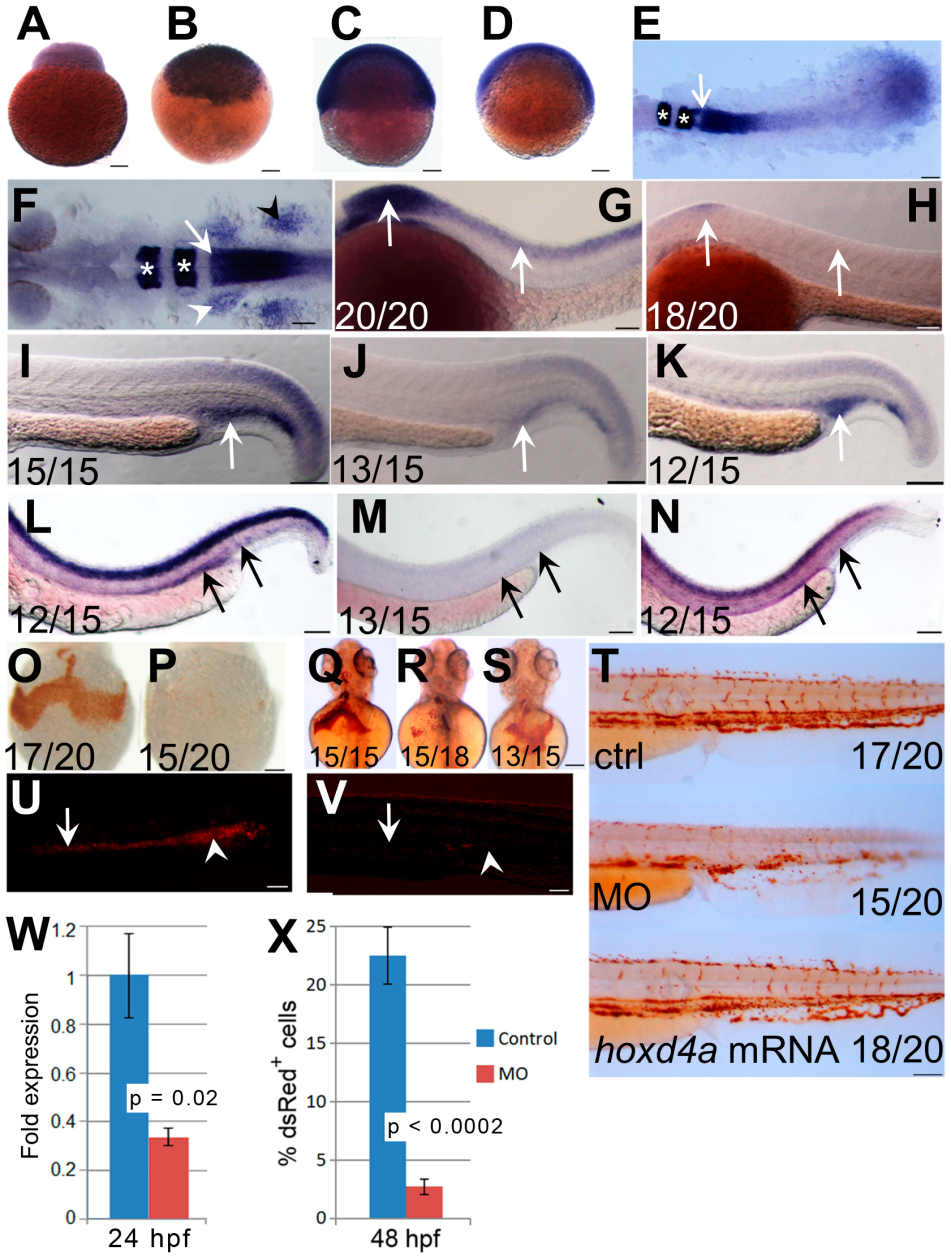
Expression pattern of *hoxd4a* and phenotype of *hoxd4a* morphants. (A–D) Detection of *hoxd4a* transcripts in early zebrafish embryos. Animal pole is to the top. (A) 1 cell. (B) 3 hpf. (C) 50% epiboly. (D) 75% epiboly. (E) Dorsal view of flat-mounted embryo at 12 hpf (5–6 somites) doubly stained for expression of *hoxd4a* and *krox20a*. The arrow indicates the anterior expression border of *hoxd4a*, while asterisks denote expression of *krox20a* in r3 and r5. Anterior is to the left. The rostral-most portion of the embryo is not captured in the image. (F) Dorsal view of a flat-mounted embryo at 26–28 hpf (>26 somites) showing *hoxd4a* expression in hindbrain, branchial arches (white arrowhead, left side only) and pectoral fin field (black arrowhead, right side only). The white arrow marks the *hoxd4a* anterior expression border in the hindbrain at the boundary between r6 and r7. Asterisks denote *krox20a* expression in r3 and r5. (G–H) Lateral views of 26–28 hpf embryos showing *hoxd4a* expression in the central nervous system (arrows) of control embryos (G) and reduced expression in *hoxd4a* morphants (H). (I–K) *hoxd4a* expression in the caudal half at 26–28 hpf as shown in lateral views, anterior to the left. *hoxd4a* expression seen in the PBI of control embryos (I, white arrow) is greatly reduced in *hoxd4a* morphants (J). Expression is rescued following co-injection with capped *hoxd4a* mRNA (K). (L–N) Results of *In situ* hybridization for *hoxd4a* at 48 hpf showing expression in the AGM and caudal vein plexus (site of future CHT) of control injected embryos (L, black arrows), greatly reduced expression in *hoxd4a* morphants (M), and rescued expression in embryos simultaneously injected with capped mRNA for *hoxd4a* (N). (O–T) Hemoglobin within RBCs revealed by o-dianisidine staining. Ventral views at 48 hpf (O,P) and 72 hpf (Q,R) show greatly reduced levels of hemoglobin in the ducts of Cuvier in *hoxd4a* morphants (P and R) vs controls (O and Q). Co-injection with capped mRNA for *hoxd4a* results in rescued RBC production (S). (T) O-dianisidine staining of the caudal half of a control larva (upper panel) and *hoxd4a* morphant (middle panel) showing overall reduction in hemoglobin levels at 72 hpf in morphants, and rescue by co-injection of capped *hoxd4a* mRNA (lower panel). (U,V) Lateral views of trunk regions of Tg(gata1:dsRed) embryos at 26 hpf, anterior to the left. The expression of dsRed within proerythroblasts is readily detected in the ICM (arrow) and PBI (arrowhead) of control-injected embryos (U) but not *hoxd4a* morphants (V). Scale bars = 100 µm. Ratios indicate the number of embryos showing the presented phenotype. (W) qRT-PCR shows an overall 3-fold reduction of *hoxd4a* expression in morphants at 26–28 hpf. Error bars = standard error. p = 0.02. (X) Quantitation by flow cytometry of dsRed-positive cells in Tg(gata1:dsRed) embryos at 48 hpf showing that morphants display an 88% reduction in RBCs relative to control-injected embryos. Error bars = standard deviation. p<0.0002.

The expression of *hoxd4a* during zebrafish development was examined by WISH. Maternal transcripts were seen at the 1 cell stage, and zygotic transcripts were readily detected from 3 hpf to 48 hpf (**Fig. 1A–G,I,L**) and in a majority of cells until at least 75% epiboly (8–9 hpf) (**Fig. 1D**). An anterior expression border in neurectoderm is visible by 10 hpf (bud stage; data not shown) with further resolution of the border between rhombomeres 6 and 7 (r6/7) by 12 hpf (**Fig. 1E** **).** As observed previously [44], [45], at 26–28 hpf *hoxd4a* is expressed in the hindbrain with an anterior border at r6/7, in neural crest migrating to the future branchial arches, and in the pectoral fin fields (**Fig. 1F**). We also observed *hoxd4a* transcripts in the PBI **(** **Fig. 1I**). At no point were *hoxd4a* transcripts detected in the PLM or ICM of control embryos. By 48 hpf, *hoxd4a* expression was apparent in the AGM and in patches in the area of the caudal vein plexus (**Fig. 1L**).

### *hoxd4a* Morphants Exhibit a Defect in Primitive Hematopoiesis

To understand the function of *hoxd4a* during zebrafish embryogenesis, we used two splice-blocking antisense MOs against either the *hoxd4a* splice donor or splice acceptor flanking the single intron interrupting the coding region. Both MOs provoked highly similar phenotypes and the results obtained with the splice-acceptor blocker are reported below. Injection of *hoxd4a* MO (8 ng) at the 1–4 cell stage resulted in severely deformed embryos that died by 7 dpf. However, at a reduced dosage of MO (4 ng), embryos exhibited grossly normal morphology up to 30 hpf but with a developmental delay of approximately 9 h. In all experiments reported below, we compared morphants and control embryos at equivalent developmental stages. In embryos of at least 26 hpf, we routinely assessed the length between the eye and the otic vesicle as suggested previously [40], as well as the size of the eye field. In some cases, we also counted somites.

Following injection of the anti-*hoxd4a* MO, *in situ* hybridization revealed a marked reduction of *hoxd4a* transcripts at 26–28 hpf (>26 somites) (**Fig. 1G–H and I–J**) confirmed by qRT-PCR (**Fig. 1W**). In particular, *hoxd4a* expression in the PBI (**Fig. 1I**) was lost in 26 hpf morphants (**Fig. 1J**). Transcripts for *hoxd4a* likewise failed to appear in the AGM and caudal vein plexus in 48 hpf morphants (**Fig. 1L–M**). Co-injection of capped *hoxd4a* mRNA rescued expression in all tissues (**Fig. 1K,N**). By contrast, the expression of *pax2.1* (a marker for intermediate mesoderm, **Fig. S1A–C vs B–D in File S1**), *nkx2.5* (a marker for cardiac mesoderm, **Fig. S1E–F in File S1**) and *myod* (a marker of paraxial mesoderm, **Fig. S1G–H in File S1**) was unaltered in the morphants, demonstrating that there was no gross defect in the overall patterning of the mesoderm.

The onset of blood circulation in zebrafish is at 25 to 26 hpf. In control-injected embryos at 48 hpf, red blood cells (RBCs) streamed normally over the yolk, through the ducts of Cuvier toward the heart, and through the dorsal aorta, posterior cardinal vein and caudal plexus. By contrast, in *hoxd4a* morphants the number of circulating RBCs was considerably reduced (**n = 96/100**) (**Videos S1, S2, S3**), but was strongly rescued by co-injection with capped mRNA for *hoxd4a* (**Video S2)**. Reduced RBC production at 48 hph (**Fig. 1O–P**) and 72 hpf (**Fig. 1Q–T**) was confirmed by staining for hemoglobin with o-dianisidine. RBC production was strongly rescued by co-injection with capped *hoxd4a* mRNA (**Fig. 1S,T**). O-dianisidine staining also revealed pooling of blood in the head and trunk in some embryos (**Fig. S1I–J in File S1; Video S3**) suggestive of vascular defects. A lack of RBCs was also apparent by observation of the heart (**Fig. S1K–L in File S1**). Apart from anemia, pericardial edema and areas of edema over the yolk sac were also observed from 36 hpf (**Fig. S1M–O in File S1**). In addition, the heart rate was also mildly but significantly slower in morphants at 26–28 hpf and 48 hpf (**Fig. S1P in File S1**), and may have affected definitive hematopoiesis (see Discussion).

As an independent assessment of the extent of erythropoiesis in *hoxd4a* morphants, we made use of the Tg(gata1:dsRed) transgenic line [38] in which the *gata1* promoter drives expression of the *Discosoma* red fluorescent protein (dsRed) in erythrocytic lineages during primitive and definitive hematopoiesis. While a strong signal was observed in the ICM and PBI of control embryos at 26 hpf, little could be detected in *hoxd4a* morphants (**Fig. 1U,V**) suggesting a deficit of erythrocytic progenitors during primitive hematopoiesis. To quantify the apparent reduction of erythrocyte numbers, we performed flow cytometry on cells isolated from Tg(gata1:dsRed) controls and morphants at 48 hpf. The results show that morphants have only 12% as many dsRed-positive cells as controls (**Fig. 1X**). Together, these findings support a marked failure of primitive hematopoiesis following *hoxd4a* knockdown.

**Figure 2:**
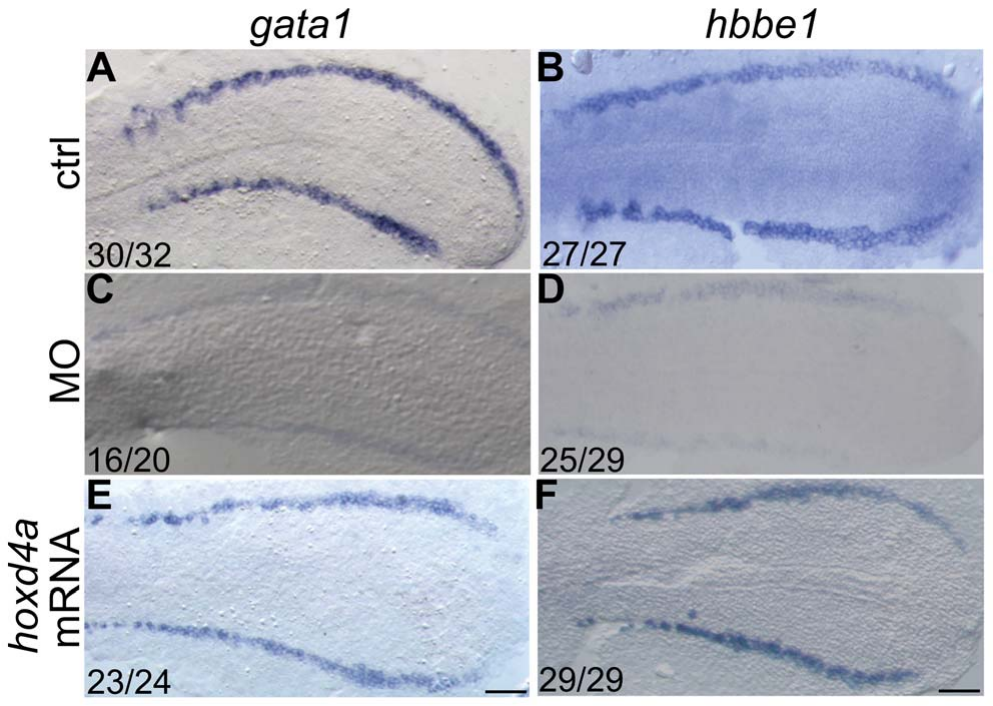
*hoxd4a* knockdown disrupts primitive hematopoiesis and is highly specific. *In situ* hybridization revealing expression at 13 hpf of erythroid lineage markers *gata1* (A,C,E) and *β embryonic globin 1* (*hbbe1*) (B,D,F). (A,B) Normal expression of *gata1* and *hbbe1* in the LPM. (C,D) Expression of *gata1* and *hbbe1* is strongly down-regulated in *hoxd4a* morphants, but rescued by co-injection of capped mRNA for *hoxd4a* (E,F). All embryos have been flat-mounted and are shown in dorsal view. Anterior is to the left. Ratios in the bottom left corner of all panels indicate the number of embryos showing the presented phenotype. ctrl, embryos injected with a non-specific morpholino. MO, embryos injected with the anti-*hoxd4a* morpholino. *hoxd4a* mRNA, embryos simultaneously injected with the anti-*hoxd4a* MO plus capped mRNA for *hoxd4a*. Scale bars equal 100 µm. All images are at the same magnification.

To assess the effect of reduced *hoxd4a* function on the genetic programme directing erythroid development, expression of the erythroid lineage-specific markers *gata1* and *β embryonic globin 1* (*hbbe1*) was analyzed by WISH. Both *gata1* and *hbbe1* transcripts were diminished in the posterior PLM at 13 hpf (∼ 8 somites) (**Fig. 2A–B** **vs C–D**) and the ICM and PBI at 26–28 hpf (**Fig. S2A–B vs C–D in File S1**), establishing a defect in primitive hematopoiesis. Co-injection of capped *hoxd4a* mRNA along with the *hoxd4a* MO significantly rescued the expression of both erythroid markers (**Fig. 2E–F** **; Fig. S2E–F in File S1**), specifically implicating *hoxd4a* in the phenotype.

### Loss of *hoxd4a* Impairs Definitive Hematopoiesis

**Figure 3:**
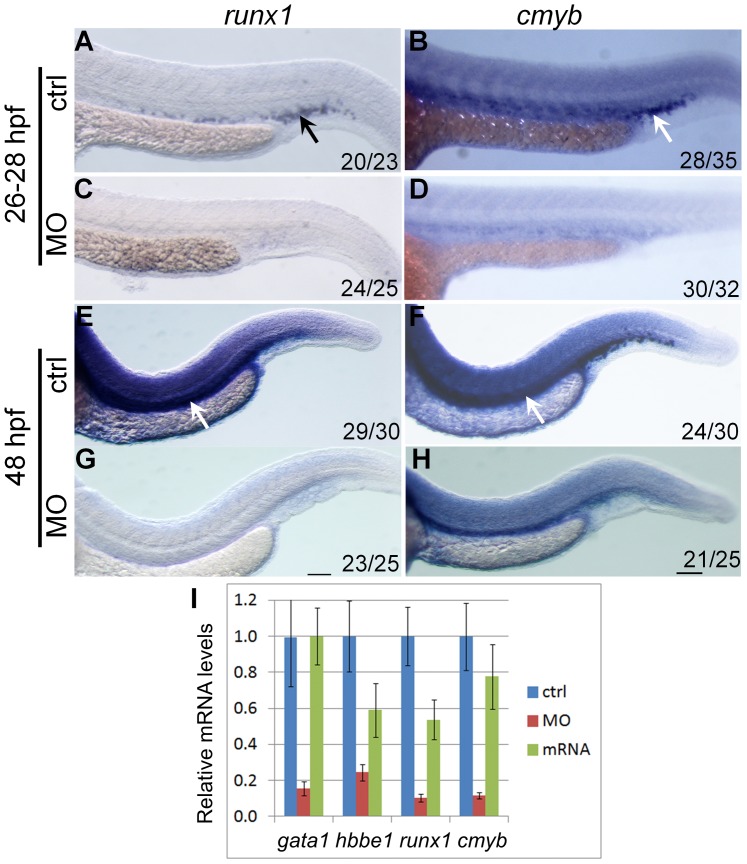
*hoxd4a* expression is required for transient and definitive hematopoiesis. *In situ* hybridization on 28 hpf (A–D) and 48 hpf (E–H) embryos showing expression of *runx1* (A,C,E,G) and *cmyb* (B,D,F,H) in presumptive HSCs arising in the PBI (arrows in A and B) and AGM (arrows in E and F). Expression of both genes was severely reduced in *hoxd4a* morphants (C,D and G,H). Ratios in the bottom right corner of images indicate the fraction of embryos showing the presented phenotype. ctrl, embryos injected with a non-specific morpholino. MO, embryos injected with the anti-*hoxd4a* morpholino. *hoxd4a* mRNA, embryos simultaneously injected with the anti-*hoxd4a* MO plus capped mRNA for *hoxd4a*. Scale bars equal 100 µm. All images are at the same magnification. (I) qRT-PCR confirms the strong depletion of hematopoietic gene expression in *hoxd4a* morphants at 26–28 hpf, and restored expression following co-injection with capped mRNA for *hoxd4a*. Samples were normalized to β-actin. Error bars indicate standard error. By comparison to controls and rescuants, the gene expression levels of all morphants were statistically different to p≤0.02 except for *gata1* control vs morphant (p = 0.04) and *hbbe1* rescuant vs morphant (p = 0.09).

Between 26 to 30 hpf, definitive hematopoiesis originates at the ventral wall of the DA, with expression of *runx1* and *cmyb* marking the HSCs [1], [3]. The expression of *runx1* and *cmyb* in *hoxd4a* morphants at 26–28 hpf (**Fig. 3A–B** **vs C-D)** and 48 hpf (**Fig. 3E–F** **vs G-H**) was severely down-regulated. The expression levels of markers of primitive and definitive hematopoiesis were quantitated by qRT-PCR and showed a strong reduction in *hoxd4a* morphants and significant rescue upon co-injection with capped *hoxd4a* mRNA (**Fig. 3I**). Thus, the knockdown of *hoxd4a* results in the impairment of both primitive and definitive hematopoietic lineages.

### Knockdown of *hoxd4a* Disrupts Endothelial Development

In addition to reduced numbers of blood cells, *hoxd4a* morphants appeared to lack a normal vasculature. At 48 hpf, blood cells circulated normally in the axial vessels and tail region in control embryos, whereas in *hoxd4a* morphants, little or no blood flow could be observed or was highly irregular with RBCs appearing to become blocked in their path through the ISVs. Blood flow was strongly rescued by co-injection with capped *hoxd4a* mRNA (**Video S2**).

**Figure 4:**
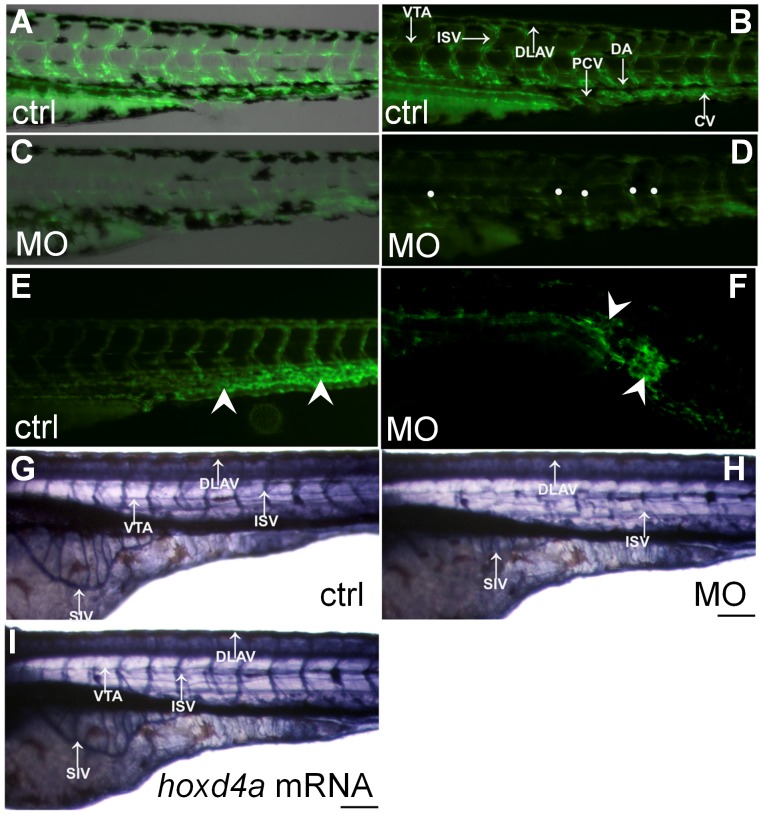
Loss of *hoxd4a* function impairs development of the vasculature. (A–D) Fluorescent images of the trunk and tail regions of Tg(*fli1*:EGFP) embryos at 48 hpf. The panels present merged bright field and fluorescent images (A,C) or fluorescent images only (B,D) The normal pattern of the vasculature (A,B) is severely disrupted in *hoxd4a* morphants (C,D) Dorsal extremities of ISV sprouts that fail to contact the DLAV are marked by white dots (D). The caudal vein plexus of control embryos (E, arrowheads) is replaced by a disorganized mass of endothelial tissue in *hoxd4a* morphants (F, arrowheads). (G–I) Alkaline phosphatase staining at 72 hpf revealing the vasculature in (G) control-injected larvae, (H) *hoxd4a* morphants, and (I) rescued larvae co-injected with capped mRNA for *hoxd4a*. Dorsal aorta (DA), posterior cardinal vein (PCV), inter-segmental vessels (ISV), caudal artery (CA), dorsal longitudinal anastomotic vessel (DLAV), caudal vein (CV) and vertebral artery (VTA). All images show lateral views, with anterior to the left and dorsal on top. Scale bars equal 100 µm.

The state of the vasculature in *hoxd4a* morphants was investigated through the use of the *fli1*:EGFP transgenic line which expresses enhanced GFP under the control of the *fli1* locus in endothelial cells [37]. While control embryos at 72 hpf exhibited normal vascular phenotypes with the formation of primary ISV joining the dorsal longitudinal anastomotic vessel (DLAV) (**Fig. 4A–B**), their morphant counterparts displayed severely impaired sprouting of ISV precursors and greatly weakened GFP signal from the region of the presumptive DA (**Fig. 4C–D**). In approximately a fifth of morphant embryos, the caudal vein plexus was replaced by an amorphous mass of endothelial tissue (**Fig. 4E–F**), while anterior bifurcation of the presumptive aortic vessel could not be observed in others (data not shown).

The effects of *hoxd4a* knockdown on vasculogenesis and angiogenesis were also assessed by exploiting the high endogenous levels of alkaline phosphatase characteristic of endothelial cells. In control embryos, intense signals revealed well-formed ISV, subintestinal vessels (SIVs) and vertebral artery (VTA), whereas such vessels were indiscernible or highly reduced in *hoxd4a* morphants (**Fig. 4G–H**). Co-injection with *hoxd4a* mRNA yielded a marked rescue of ISV, SIV and VTA (**Fig. 4I**). The pooling of blood that we observed might be attributable to hemorrhaging resulting from these defects in vascular development (**Fig. S1I–J in File S1; Video S3**). Similar hemorrhaging has been observed in *fli1* morphants [46]. Together, our results confirm profound defects in vasculo- and angiogenic processes in *hoxd4a* morphants.

**Figure 5:**
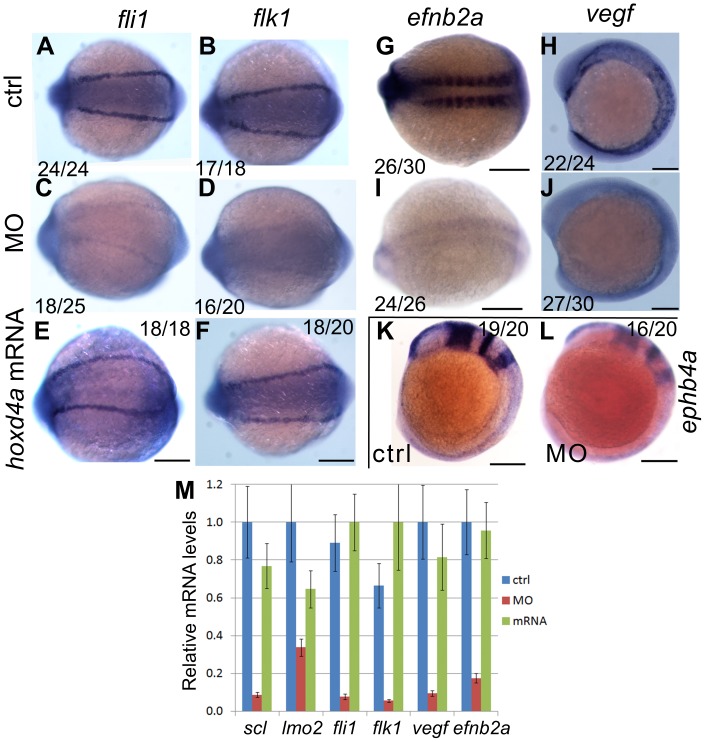
Knockdown of *hoxd4a* disrupts the endothelial programme in zebrafish embryos. *In situ* hybridization at 13 hpf revealing expression of pan-endothelial markers *fli1* and *flk1* in control-injected embryos (A,B), *hoxd4a* morphants (C,D), and rescued embryos co-injected with anti-*hoxd4a* MO and capped mRNA for *hoxd4a* (E,F). (G,I) Expression of the marker of arterial identity *efnb2a* in controls (G) and *hoxd4a* morphants (I). (H,J) Expression of the endothelial inducer *vegf* in controls (H) and *hoxd4a* morphants (J). (K,L) Expression of the venous marker *ephb4a* in controls (K) and *hoxd4a* morphants (L). Ratios indicate the fraction of embryos showing the presented phenotype. All images show dorsal views with anterior to the left except H, J, K and L which are lateral views with anterior to the left. Scale bars equal 100 µm. (M) qRT-PCR results showing depletion at 26–28 hpf of angioblast and vascular gene expression in *hoxd4a* morphants and rescue by co-injection of capped mRNA for *hoxd4a*. Samples were normalized to β-actin. Error bars indicate standard error. By comparison to controls and rescuants, the gene expression levels of all morphants were statistically different to p≤0.02 except for *lmo2* control vs morphant (p = 0.04) and *lmo2* rescuant vs morphant (p = 0.05).

To evaluate the effect of *hoxd4a* knockdown on genetic programs directing vasculogenesis and angiogenesis, we performed *in situ* hybridization for the endothelial markers *fli1* and *flk1.* Both genes were significantly reduced in the LPM of *hoxd4a* morphants at 13 hpf (**Fig. 5A–B** **vs C–D**), and in the major trunk vessels and ISVs at 26 hpf (**Fig. S3A–B vs C–D in File S1**), implying that *hoxd4a* morphants are defective in axial vasculature. Co-injection of capped mRNA for *hoxd4a*, rescued both *fli1* and *flk1* expression, establishing a specific role for *hoxd4a* in the altered gene expression pattern (**Fig. 5E–F** **; Fig. S3E–F in File S1**).

Early in development, a common primary blood vessel forms at the midline, partly under the influence of VEGF secreted by ventral somitic tissue. The DA and PCV derive from this common primary vessel through a process of ventral sprouting dependent on *efnb2a* and *ephb4a* [2]. We observed an amorphous mass of tissue in the region of the caudal vein plexus at 48 hpf (**Fig. 4F**), a site of *hoxd4a* expression (**Fig. 1H**) and a failure of anterior aortic bifurcation (data not shown), suggesting defects in primary vessel formation. We therefore assessed the expression of *vegf*, the arterial marker *efnb2a* and the venous marker *ephb4a*. At 13 hpf, the expression of *efnb2a* and *vegf* was significantly reduced in the morphants, implicating defective artery specification (**Fig. 5G–H** **vs I–J**). Likewise, the expression of the vein marker *ephb4a* was reduced at 13 hpf (**Fig. 5K–L**). The expression of *efnb2a* remained attenuated at 26–28 hpf, but that of *ephb4a* had recovered (**Fig. S3 G–J in File S1**). Results for *fli1*, *flk1*, *vegf* and *efnb2a* were quantified by qRT-PCR on RNA extracted from 26–28 hpf embryos. The results confirm the decreased expression of these genes in *hoxd4a* morphants, and the ability of co-injected mRNA for *hoxd4a* to rescue their expression (**Fig. 5M**).

### A Role for *hoxd4a* in Hemangioblast Formation through Regulation of *meis1.1*

**Figure 6:**
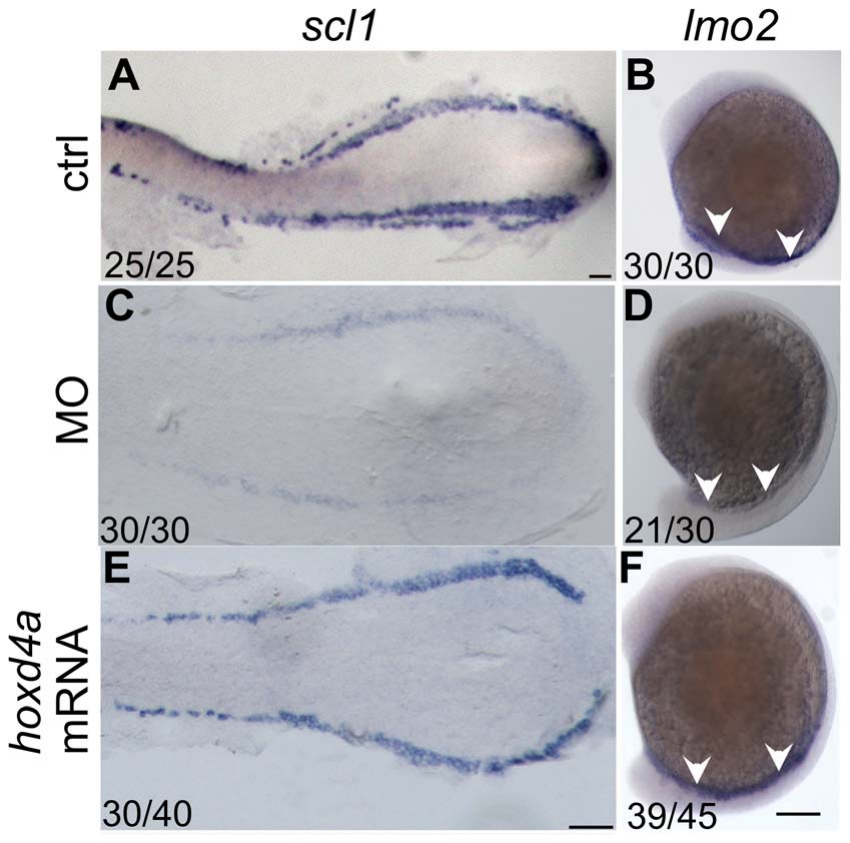
*hoxd4a* is required for hemangioblast formation. (A,B) Normal expression at 13 hpf of posterior hemangioblast markers *scl1* (A) and *lmo2* (B) in the PLM. (C,D) Expression of these markers is greatly reduced in *hoxd4a* morphants, but is rescued by co-injection of capped mRNA for *hoxd4a* (E,F). Ratios indicate the fraction of embryos showing the presented phenotype. Anterior is to the left. A, C and E are dorsal views of flat-mounted specimens while B, D and F are lateral views. Scale bars equal 100 µm. A is at a lower magnification than C and E.

The hemangioblast is the common precursor to the blood and endothelial lineages and is defined by early expression of such genes as *fli1*, *scl1* and *lmo2* in both the ALM and PLM, and *gata5* in the ALM only [1], [3], [46], [47]. The severe defects in both blood and endothelial lineages in *hoxd4a* morphants suggested that the hemangioblast itself may be compromised. To assess this idea, the expression of hemangioblast markers *scl1* and *lmo2* was analyzed by *in situ* hybridization. Significant down-regulation of both genes was observed in morphants at 13 hpf (**Fig. 6A–B** **vs C–D**). The impaired expression of *scl* and *lmo2* persisted at 26 hpf (**Fig. S4A–B vs C–D in File S1**), suggesting that their later independent functions in definitive hematopoiesis were also compromised. Co-injection of capped *hoxd4a* mRNA rescued *scl* and *lmo2* expression at 13 hpf (**Fig. 6E–F**) and 26 hpf (**Fig. S4E–F in File S1**).

**Figure 7:**
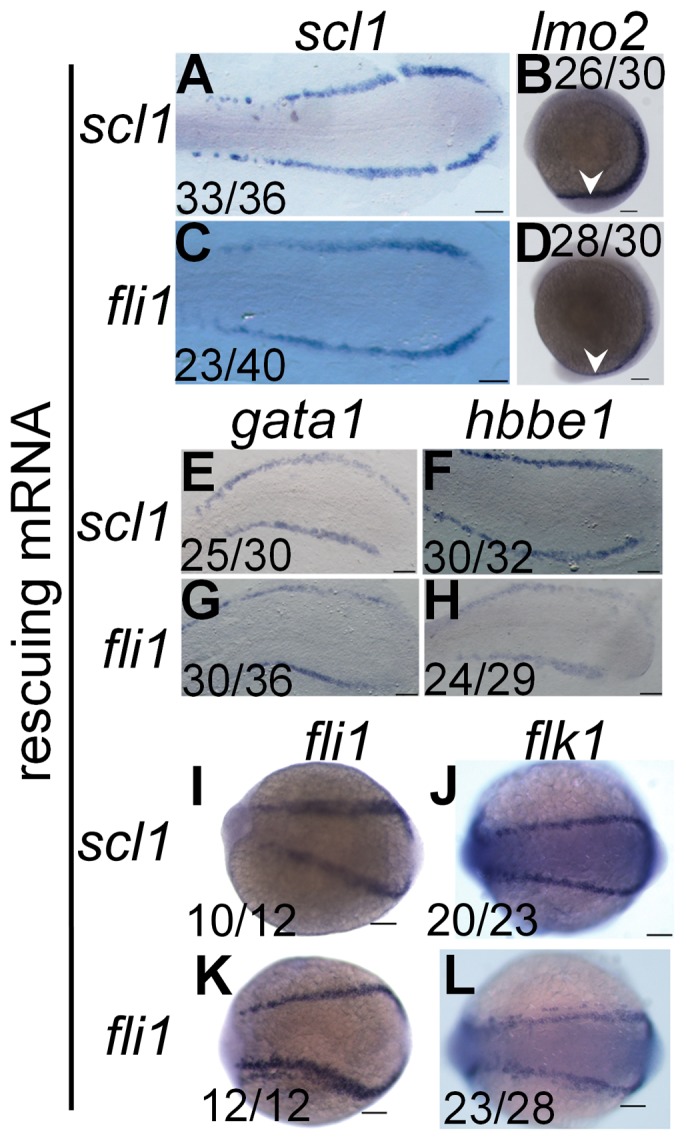
*scl1* and *lmo2* act downstream of *hoxd4a* to direct formation of the hemangioblast. All images are of *hoxd4a* morphants at 13 hpf. *In situ* hybridization was performed to detect expression of *scl1* and *lmo2* (A–D), *gata1* and *hbbe1* (E–H) and *fli1* and *flk1* (I–L). To test for rescue of gene expression, embryos were co-injected with capped mRNA for either *scl1* or *fli1* as indicated on the left. Ratios indicate the fraction of embryos showing the presented phenotype. Scale bars equal 100 µm.

To establish the hierarchy between these key regulators, the *hoxd4a* MO was co-injected with capped mRNA for either *scl* or *fli1*. The expression of *scl* and *lmo2* was rescued in both cases (**Fig. 7A–B** **vs C-D; Fig. S5A–B vs C–D in File S1**), placing the action of *hoxd4a* above these early acting effectors of hemangioblast specification [3], [46].

Consistent with the above findings, expression of *gata1* and *hbbe1* at 13 and 26 hpf was rescued by co-injection of *scl* and *fli1* mRNA (**Fig. 7E–H** **; Fig. S5E–H in File S1**), though rescue by *fli1* at 13 hpf was incomplete. Expression of the vasculogenic markers *fli1* and *flk1* was likewise rescued by *scl* and *fli1* mRNA at 13 hpf (**Fig. 7I–L**) and 26 hpf (**Fig. S5I–L in File S1**). Together, these results reveal that *hoxd4a* occupies a very high position in the transcriptional hierarchy governing hemangioblast formation or function.

A number of studies have demonstrated major regulatory roles for Hox transcription factors in hematopoietic cell fate decisions [11]–[13], [22]. HOX proteins interact with other DNA-binding cofactors including TALE homeoproteins of the MEIS and PBX families, and *Meis* homologs have been implicated in the earliest stages of hematopoiesis and vasculogenesis in mice and zebrafish [18]–[20]. We therefore examined the expression of *meis1.1* in *hoxd4a* morphants.

**Figure 8:**
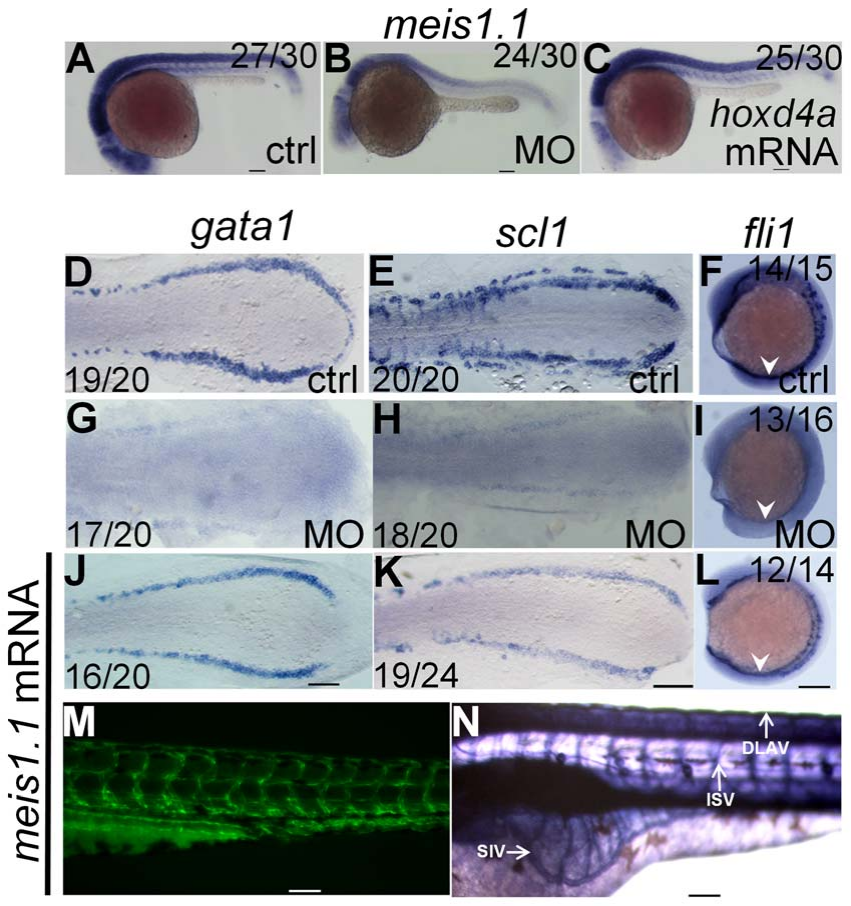
*meis1.1* is down regulated in *hoxd4a* morphants and *meis1.1* mRNA rescues hematopoietic and vasculogenic gene expression in *hoxd4a* morphants. Expression of *meis1.1* in control (A), morphant (B) and *hoxd4a*-rescuant (C) embryos at 26–28 hpf. (D–I) At 13 hpf, normal expression of *gata1*, *scl1* and *fli1* (D–F) is reduced in *hoxd4a* morphants (G–I), but rescued upon co-injection with capped mRNA for *meis1.1* (J–L). (M,N) Rescue of vascular patterning in *hoxd4a* morphants by co-injection with capped mRNA for *meis1.1* as visualized in Tg(fli1:EGFP) transgenics at 48 hpf (M) and by alkaline phosphatase staining at 72 hpf (N). Scale bars equal 100 µm.

At 13 hpf, *meis1.1* expression was significantly reduced in the PLM of *hoxd4a* morphants (**Fig. S6E–F in File S1**), while at 26–28 hpf *meis1.1* expression was reduced throughout the embryo (**Fig. 8A–B** and below) and could be rescued with capped *hoxd4a* transcripts (**Fig. 8C**). These results strongly suggested that one of the primary causes of hematopoietic and vasculogenic defects in *hoxd4a* morphants may be the loss of *meis1.1* function. To test this notion, we attempted to rescue the expression of the genetic markers of hemangioblast formation, primitive hematopoiesis and vasculogenesis (*scl, gata1* and *fli1*) in *hoxd4a* morphants by co-injection with mRNA for *meis1.1*. All three markers were strongly rescued at 13 hpf (**Fig. 8D–L**). Likewise, restoration of *meis1.1* rescued the normal development of the vasculature including the DA, ISV, DALV and SIV as revealed in *fli1*:EGFP transgenics (**Fig. 8M**) and following visualization of the vasculature with alkaline phosphatase (**Fig. 8N**). The circulation is also largely restored in these embryos, especially robust in the DA, though less evident in the return flow through the caudal vein plexus and PCV (**Video S4**).

Along with *cloche* [7], the *cdx1* and *cdx4* genes are among the earliest effectors of the hemangioblast lineage [9], [10]. Doubly deficient *cdx1*/*cdx4* embryos lack midline vasculature and show a complete failure of hematopoiesis [9]. In *hoxd4a* morphants, *cdx4* expression is unaltered at 13 hpf (**Fig. S6A–B in File S1**) and 26 hpf (**Fig. S7Q–R in File S1)**. Moreover, injection of capped *cdx4* mRNA fails to rescue defects of hematopoiesis or the vasculature in *hoxd4a* morphants (data not shown). We conclude that *hoxd4a* acts downstream or parallel to *cdx1*/*cdx4* but upstream or parallel to *meis1.1*.

**Figure 9:**
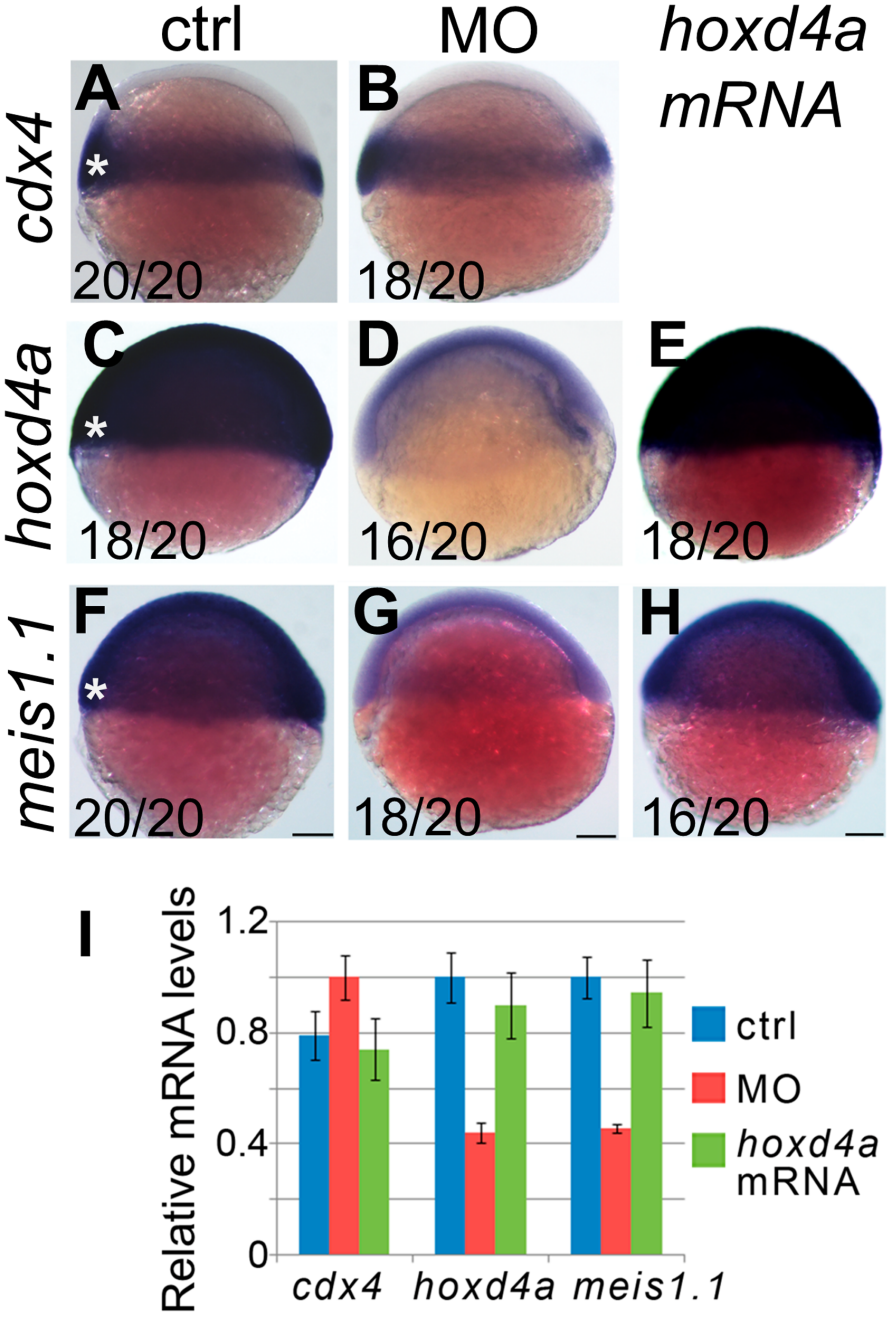
Expression of *meis1.1,* but not *cdx4*, is impaired in *hoxd4a* morphants at the shield stage. Expression of *cdx4* (A,B), *hoxd4a* (C–E) and *meis1.1* (F–H) in control (A,C,F), *hoxd4a* morphants (B,D,G) and rescuants injected with *hoxd4a* mRNA (E,H) observed at the shield stage. Asterisks denote the ventral-most mesoderm fated to give rise to hemangioblast in addition to unipotential hematopoietic and angiogenic progenitors. Ratios indicate the fraction of embryos showing the presented phenotype. Scale bars equal 100 µm. (I) qRT-PCR was used to quantitate relative mRNA levels in controls, morphants and rescuants as indicated. Error bars present the standard error. Decreased expression of *hoxd4a* and *meis1.1* in *hoxd4a* morphants is significantly lower than both controls and rescuants to p≤0.02.

We then asked whether *cdx4*, *hoxd4a* and *meis1.1* are ever temporally and spatially co-expressed so as to directly implement this hierarchy in a cell-autonomous fashion. Prior fate-mapping studies have established that precursors to the PLM hemangioblasts arise from the ventral-most presumptive mesoderm of shield-stage embryos [5], [35], [36], a tissue in which *hoxd4a* is expressed (**Fig. 1C**). *In situ* hybridization on shield-stage embryos showed strong overlapping expression of *cdx4*, *hoxd4a* and *meis1.1* in ventral-most mesoderm (**Fig. 9A,C,F**). Importantly, knockdown of *hoxd4a* provoked a reduction in *hoxd4a* and *meis1.1* expression in this same tissue (**Fig. 9D,G**). By contrast, *cdx4* expression was unaffected (**Fig. 9B**). Importantly, co-injection of *hoxd4a* mRNA restored *hoxd4a* transcript levels and rescued the expression of *meis1.1* (**Fig. 9E,H**). qRT-PCR on mRNA from controls, *hoxd4a* morphants and rescuants at the shield-stage validated results from *in situ* hybridization (**Fig. 9I**). The co-expression of *hoxd4a* with *cdx4* and *meis1.1* in tissue fated to give rise to PLM hemangioblasts, and the control of *meis1.1* expression by *hoxd4a* in this same tissue, strongly suggests that the failure to form PLM hemangioblasts in *hoxd4a* morphants is due to defects initiated at the shield stage.

**Figure 10:**
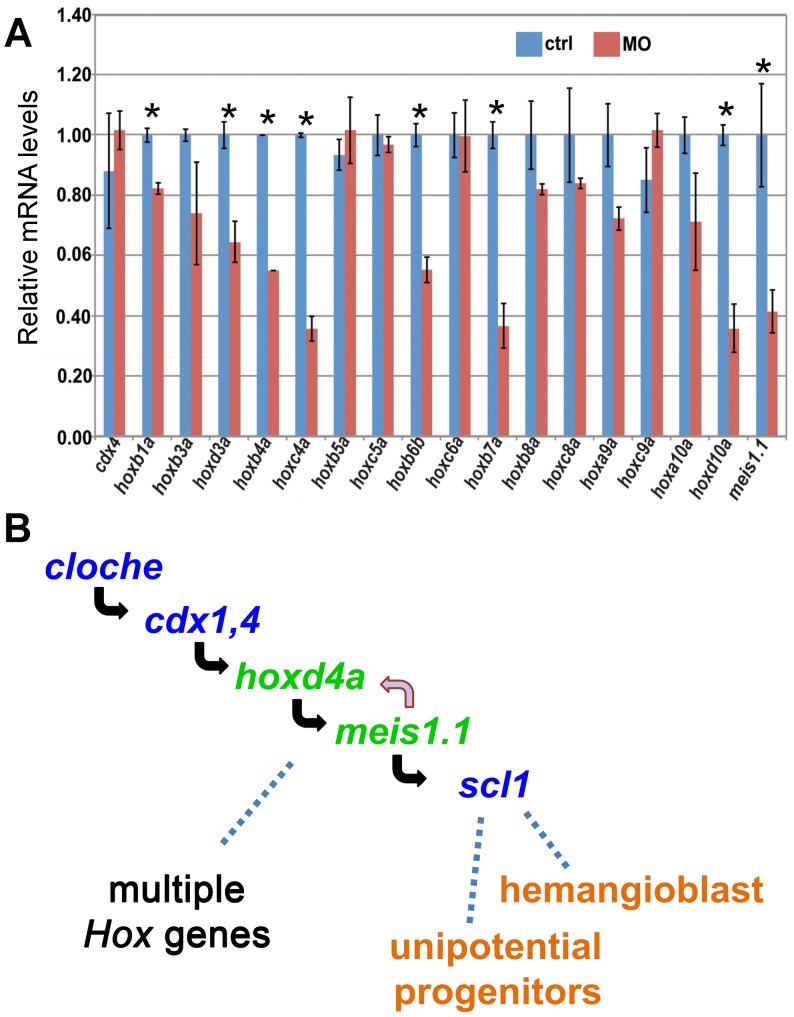
*cdx4*, *meis1.1* and Hox gene expression in *hoxd4a* morphants, and a genetic pathway for specification of hemangioblasts and unipotential stem cells. (A) qRT-PCR showing decreased expression of *meis1.1* and many, but not all, *hox* genes in *hoxd4a* morphants at 26–28 hpf. By contrast, *cdx4* levels are unchanged. Samples were normalized to *β-actin*. Error bars indicate standard error. All pairs marked with an asterisk meet statistical significance (p≤0.02). (B) Based on the results presented here and those in the literature, we propose a pathway in which *hoxd4a* and *meis1.1* occupy sequential steps downstream of *cloche* and *cdx1/4* in a genetic programme leading to the specification of hemangioblasts and unipotential angiogenic and hematopoietic stem cells. The effects of *hoxd4a* knockdown may be magnified through positive cross-regulatory interactions with *meis1.1*. The observed effects on the expression of multiple Hox genes could be due to the direct action of *hoxd4a* and *meis1.1*. Non-exclusively, *cdx1* and *cdx4* may act in conjunction with *hoxd4a* and *meis1.1* in a feed-forward type of mechanism to regulate one or more of these same Hox genes with widespread consequences for vasculogenesis, angiogenesis and hematopoiesis at all levels.

Members of the MEIS family regulate Hox gene expression in both invertebrates and vertebrates [14], [48], [49]. Both gene families have likewise been implicated in hematopoietic and angiogenic processes [13], [18]–[20], [22]. We therefore wished to determine whether knockdown of *hoxd4a* and subsequent reduction in *meis1.1* function might have provoked Hox gene deregulation. We assessed the expression of multiple Hox genes following *hoxd4a* knockdown at 26–28 hpf by quantitative RT-PCR (**Fig. 10A**). More than half of the Hox genes tested showed significant down-regulation, including *hoxb4a*, *hoxb6b*, *hoxb7a* and *hoxa9a*, all of which have been strongly implicated in hematopoietic processes. Likewise, *hoxd3a*, whose ortholog has been shown to promote angiogenesis in the mouse [50], [51], was also down-regulated. Consistent with results from *in situ* hybridization, *cdx4* expression was unaffected. *In situ* hybridization on a number of these Hox genes confirmed decreased expression at 13 and 26–28 hpf (**Fig. S7 in File S1**). These results validate the suggestion that the early loss of *meis1.1* function leads to the deregulation of several, though not all, Hox genes, thereby providing a fuller explanation for how the initial impairment of a single Hox gene, *hoxd4a*, can lead to massive defects in hematopoiesis, vasculogenesis, and angiogenesis.

## Discussion

This study places *hoxd4a* near the top of a regulatory cascade directing hematopoiesis, vasculogenesis and angiogenesis in zebrafish embryos (**Fig. 10B**). This unexpected role of *hoxd4a* is at least partly due to its positive regulation of *meis1.1*, and is likely to have both direct and indirect consequences. Most directly, decreased *hoxd4a* function leads to reduced expression of *meis1.1* in the ventral-most presumptive mesoderm of the shield-stage embryo, a tissue fated to produce the hemangioblast in addition to unipotential erythrocytic and endothelial progenitors [5], [35], [36]. A few hours later, hemangioblast markers are severely reduced in the PLM, leading us to propose that *hoxd4a* and *meis1.1* loss of function at the shield stage interferes with the specification of hemangioblasts and unipotential progenitors. Less directly, *hoxd4a* knockdown leads to massive deregulation of *Hox* gene expression, including a number of *Hox* genes previously implicated in these processes such as *hoxb4a*, *hoxb6b*, *hoxb7a* and *hoxa9a* [9], [10].

In support of this model, injection of capped mRNA for either *hoxd4a* or *meis1.1* rescues essentially all aspects of the knockdown phenotype. It is likely that much of the effect of the loss of *meis1.1* expression follows from the impaired function of MEIS-PBX and MEIS-PBX-HOX transcriptional complexes [14], [20], [52], though functions of MEIS that are independent of HOX or PBX should also be affected.

Three reports describe the effects of *meis1.1* loss of function on hematopoiesis and vasculogenesis in zebrafish embryos [18]–[20]. Only Ouwehand and co-workers [18] observed, as we do, defects in both processes. Suzuki and co-workers [19] observed defects in the vasculature, heart edema and weakened heartbeat resulting from decreased *meis1.1* function, and implicated a downstream impairment in *flk1* and *vegf* expression, consistent with our findings. Waskiewicz and co-workers [20] found that loss of *meis1.1* function caused severe defects in erythropoiesis, but did not observe vasculature defects nor detect alterations in either *flk1* or Hox expression. Together, these results suggest that the complement of defects we observe in this study are likely to exceed what can be explained by a simple loss of *meis1.1* function. A full account of the phenotypes reported here will likely have to consider the roles of *meis1.1*, *hoxd4a* itself, and the downstream effects on the expression of multiple *Hox* genes.

The phenotype we report here is in some ways closer to that reported for *cdx1* plus *cdx4* double loss-of-function embryos, including severe hematopoietic (both primitive and definitive) and vasculogenic defects, and impaired expression of multiple *Hox* genes [9], [10]. By contrast, *Hox* gene expression is not affected in *meis1.1* or *meis3* morphants [20], [53]. However, we do not observe significant changes in *cdx4* expression in *hoxd4a* morphants, and the *hoxd4a* morphant phenotype cannot be rescued by the injection of capped mRNA for *cdx4*. It may be that *hoxd4a* is an immediate downstream effector of *cdx1/4* function. Alternatively, *hoxd4a* may act in parallel with *cdx1* and *cdx4* such that their function, but not expression, is compromised. Such a mechanism is implied by recent studies showing that products of murine CDX1 and HOXD4 form functional heterodimers [54]. A direct role for *cdx1* and *cdx4* in definitive hematopoiesis could not be established with certainty due to major effects in the development of the dorsal aorta in doubly deficient embryos [9]. By contrast, *hoxd4a* morphants do form distinctly separate DA and PCV, and these vessels are open to circulation. Nonetheless, we did observe impaired expression of the arterial marker *efnb2a*, suggesting that the DA may not be appropriately specified in *hoxd4a* morphants, an event that could lead to defective HSC formation. The mildly reduced heart rate of morphants may contribute to decreased *efnb2a* expression and impaired specification of HSCs due to sub-optimal levels of nitrous oxide (NO) signaling [55]. This raises the possibility that the observed impairment of definitive hematopoiesis is a secondary event, and we are therefore cautious in assigning a direct role for *hoxd4a* and *meis1.1* in this process. Nonetheless, depressed NO signaling cannot explain all of the defects noted here, since the reduced *flk1* expression that we observe at 13 and 26 hpf is not an expected consequence of decreased heart rate [56], [57]. In addition, *hoxd4a* morphants show reduced *efnb2a* expression in tissues that should not be affected by NO levels consistent with a direct role for *hoxd4a* or *meis1.1* and similar observations in *meis1.1* morphants [18], [19].

Some of the phenotypic defects observed here can be explained by effects on relatively early embryonic events, such as impaired formation and function of hemangioblast precursors, as indicated by the loss of hemangioblast markers *scl*, *lmo2* and *fli1*, and the ability of the capped mRNAs for these genes to rescue development. However, other aspects of the phenotype may represent later and considerably more downstream functions. For example, decreased expression of *efnb2a* points to a defect in arterial specification downstream of hemangioblast formation and function, as has been noted in *meis1.1* morphants [18], [19]. Likewise, much of the vasculogenic and angiogenic defects could be explained by the impairment of the downstream roles of *fli1, vegf* and *flk1* in these processes. The *vegf* gene is also known to play a role in the initiation of hematopoiesis [4], and its downregulation is therefore likely to contribute to many aspects of the phenotype reported here.

Expression of *gata1* commits hematopoietic precursors to erythropoiesis during primitive hematopoiesis in the ICM. Later in development, *runx1* and *cmyb* are required for myeloid development during definitive hematopoiesis in the equivalent of the AGM [1], [3]. Following *hoxd4a* knockdown, decreased expression of *scl* and *lmo2* is likely to contribute to a failure in the induction of *gata1* and defective primitive hematopoiesis as evidenced by a loss of expression of *hbbe1*; but loss of *scl* and *lmo2* function would also account for the observed defect in *runx1* and *cmyb* expression and the subsequent failure of definitive hematopoiesis. Interestingly, we observe the expression of *hoxd4a* at 26–28 hpf in the PBI and at 48 hpf in the AGM and caudal vein plexus. The caudal vein plexus is the site of the future CHT [4], an early site of definitive hematopoiesis, functionally equivalent to the fetal liver of mice [4], [6]. Thus, *hoxd4a* might play a direct role in primitive and definitive hematopoiesis apart from its indirect role through *meis1.1* earlier in development. Both early and later functions of *meis1.1* in hematopoiesis and vasculogenesis are also likely, given very early effects on the expression of hemangioblast markers and later expression of *meis1.1* in the ICM as described by others [19], [58]. Multiple sites of action along a pathway leading from hemangioblast specification to vasculogenesis, angiogenesis and primitive and definitive hematopoiesis could also help to explain why epistasis experiments give apparently conflicting results. For example, one report places *meis1.1* downstream of *scl* for the induction of primitive hematopoiesis [18], whereas our own work places *meis1.1* upstream of *scl* at an earlier time point during hemangioblast specification.

Hox gene expression in vertebrates is implemented in two phases, an early phase in presumptive mesoderm around gastrulation and a late phase in the body proper [59]–[65]. During gastrulation in *Xenopus*, timed interactions between the organizer and non-organizer mesoderm induce Hox expression and pattern the AP axis [59], while impaired Hox gene expression can alter the timing of ingression through the primitive streak in the gastrulating chick [65]. Although the widespread deregulation of late-phase Hox gene expression seen after *hoxd4a* knockdown could be attributed to defects in gastrulation, two observations argue against this. First, not all the Hox genes we tested were affected, an observation inconsistent with a global gastrulation defect. Second, our demonstration that the expression of markers of paraxial mesoderm (*myod*), cardiac mesoderm (*nkx2.5*) and intermediate mesoderm (*pax2.1*) was unaffected does not support a major impairment of gastrulation movements. Nonetheless, our findings emphasize the importance of Hox gene function in the early peri-gastrulation phase [61]–[65].

Mice lacking *Hoxd4* function are viable and fertile, and display defects of the anterior vertebral skeleton [33], [34]. Although an extensive analysis of vasculogenesis and hematopoiesis has not been undertaken in *Hoxd4* null mice, their viability suggests that there is no severe impairment of these processes. By contrast, mutation of the paralogous *Hoxb4* gene does lead to hematopoietic defects and some loss of viability, though these mice are still able to complete embryogenesis and many survive to adulthood [17], [34], [66]–[68]. In striking contrast to the mouse, we show here that loss of *hoxd4a* function has severely deleterious consequences for hematopoiesis, vasculogenesis and angiogenesis. We suggest that this may be due to a dependency of *meis1.1* expression on *hoxd4a* function that was either acquired by teleosts (or simply acquired by zebrafish) or lost in mammals. It may also be that while *Hoxb4* has the predominant role in these processes in mammals, evolution has selected for *hoxd4a* to take on these functions in teleosts. This situation may be partly analogous to that of the *midkine* orthologs in mice and zebrafish; the two teleost *midkine* genes are strongly expressed in the adult brain, whereas the mouse ortholog is not [69], implying the acquisition or loss of function in the course of evolution.

## Supporting Information

File S1 **Supporting information.** Contains supplementary Figures S1 to S7 and supplementary Tables S1 and S2.
(PDF)

Figure S1 **Patterning and morphology in** ***hoxd4a*** **morphants.** (A–H) Knockdown of *hoxd4a* does not perturb overall patterning of the mesoderm. (A–D) Expression of *pax2.1* shows that intermediate mesoderm forms and matures normally in *hoxd4a* morphants at 13 hpf (B) and 26–28 hpf (D) vs controls (A,C). (E,F) *nkx2.5* expression in the precardiac lateral plate mesoderm at 13 hpf is normal in control (E) and morphant embryos (F). (G,H) *myod* expression in paraxial mesoderm is normal in control (G) and morphant embryos (H). Images in C, D, G and H are lateral views with anterior to the left. A, B, E and F show dorsal views with anterior to the top (A,B) or left (E,F). (I to O) Lateral views of control and *hoxd4a*-MO-injected larvae at 72 hpf. (I,J) Staining of hemoglobin with o-dianisidine reveals areas of hemorrhage such as in the head (I, arrowheads) and trunk (J, arrowheads) in some *hoxd4a* morphants. (K,L) Control larvae (K) but not *hoxd4a* morphants (L) show abundant RBCs passing through the heart (arrowheads). (M–O) Unlike control larvae (M), *hoxd4a* morphants display pericardial edema and edema over the adjacent yolk (N,O, arrowheads). Scale bars equal 100 µm. (P) The heart rate in morphants at 26–28 and 48 hpf was mildly reduced, but in a statistically significant manner as determined by unpaired Student’s t test (p<0.0001). Error bars give standard deviation.
(TIF)

Figure S2 **Reduced expression of markers of primitive hematopoiesis** ***gata1*** **and** ***β embryonic globin*** **(** ***hbbe1*** **) in** ***hoxd4a*** **morphants.** WISH in control and morphant embryos at 26–28 hpf showing the expression of *gata1* (A,C,E) and *hbbe1* (B,D,F) in the ICM and PBI (white arrowheads). Normal expression of *gata1* and *hbbe1* (A,B) is severely reduced in *hoxd4a* morphants (C,D) and rescued by co-injection with capped mRNA for *hoxd4a* (E,F). All images are lateral views with anterior to the left. ctrl, embryos injected with a non-specific morpholino. MO, embryos injected with the anti-*hoxd4a* morpholino. *hoxd4a* mRNA, embryos simultaneously injected with the anti-*hoxd4a* MO plus capped mRNA for *hoxd4a*. Scale bars equal 100 µm. All images are at the same magnification.
(TIF)

Figure S3 **Reduced expression of markers of angiogenesis and venous specification in** ***hoxd4a*** **morphants.** WISH in control and morphant embryos at 26–28 hpf showing the expression of *fli1* (A,C,E) and *flk1* (B,D,F). Normal expression of *fli1* and *flk1* (A,B) is severely reduced in *hoxd4a* morphants (C,D) and rescued by co-injection with capped mRNA for *hoxd4a* (E,F). White or black dots denote the tips of dorsally sprouting ISVs. Relative to controls (G,H), the expression of the arterial marker *efnb2a* is reduced in morphants at 26–28 hpf (I), while the venous marker *ephb4a* in morphants has recovered (J). Scale bars equal 100 µm.
(TIF)

Figure S4 **Reduced expression of** ***scl*** **and** ***lmo2*** **in** ***hoxd4a*** **morphants at 26–28 hpf.** (A–J) Expression analysis of *scl* (A,C,E) and *lmo2* (B,D,F) at 26–28 hpf. Normal expression of *scl* and *lmo2* (A,B) is severely reduced in *hoxd4a* morphants (C,D) and rescued by co-injection with capped mRNA for *hoxd4a* (E,F) All images present lateral views with anterior to the left and dorsal on top. Scale bars equal 100 µm. All images are at the same magnification.
(TIF)

Figure S5 ***scl1*** **and** ***fli1*** **act downstream of** ***hoxd4a*** **to direct formation of the hemangioblast.** All images are of *hoxd4a* morphants at 26–28 hpf previously injected with capped mRNAs for either *scl1* or *fli1* as indicated on the left. WISH was performed to detect expression of *scl1* and *lmo2* (A–D), *gata1* and *hbbe1* (E–H) and *fli1* and *flk1* (I–L). Scale bars equal 100 µm. All images are at the same magnification.
(TIF)

Figure S6 **Knockdown of** ***hoxd4a*** **results in decreased expression of** ***meis1.1*** **but not** ***cdx4*** **at 13 hpf (∼8 somites).** (A–F) Expression of *cdx4* (A,B), *hoxd4a* (C,D) and *meis1.1* (E,F) in control (A,C,E) and *hoxd4a* morphants (B,D,F) at the shield stage. The white arrowheads in C and D denote the *hoxd4a* anterior expression boundary in the hindbrain. Scale bars equal 100 µm. All images are at the same magnification.
(TIF)

Figure S7 **The expression of multiple** ***hox*** **genes is reduced at 26–28 hpf in** ***hoxd4a*** **morphants.** Images are dorsal views (A–H) and lateral views (I–P) of embryos taken through *in situ* hybridization for the indicated *hox* genes. Relative to control embryos (A,C,E,G,I,K,M,O), *hox* gene expression is reduced in *hoxd4a* morphants (B,D,F,H,J,L,N,P). All embryos were simultaneously probed for *krox20a* expression in r3 and r5 as in Figure 1C. (Q–R) *cdx4* expression is unchanged in control (Q) and *hoxd4a* morphants (R) at 26–28 hpf. Scale bars equal 100 µm. All images are at the same magnification.
(TIF)

Table S1 **Primers used for cDNA cloning.**
(TIF)

Table S2 **Primers for quantitative RT-PCR.**
(TIF)

Video S1 **Anterior blood flow in control and** ***hoxd4a*** **morphant embryos.** Lateral view focusing on the anterior half of a 48 hpf embryo including the region of the heart, future branchial arches and yolk of control embryos and *hoxd4a* morphants. In particular, note robust streaming of blood cells through the ducts of Cuvier over the yolk in the control, but an almost complete absence of circulation in the *hoxd4a* morphant.
(7Z) Click here for additional data file.

Video S2 **Trunk blood flow in control,** ***hoxd4a*** **morphant and rescued embryos.** Lateral view of the trunk at 48 hpf in a control, *hoxd4a* morphant and rescuant previously injected with capped mRNA for *hoxd4a*. Circulation is vigorous in control and rescuant embryos, with abundant RBCs flowing caudally along the DA, streaming dorsally through the ISVs, caudally through the DLAV, and rostrally through the caudal vein and PCV. By contrast, the number of blood cells is greatly reduced in morphants and blood cells are unable to transit the truncated ISVs.
(7Z) Click here for additional data file.

Video S3 **Tail blood flow in control and** ***hoxd4a*** **morphant.** Lateral view of the tail region in control and *hoxd4a* morphant embryos at 48 hpf. Control embryos display vigorous blood flow through the DA, ISVs, DLAV and caudal vein plexus. By contrast, morphants show a highly reduced blood cell count with individual blood cells moving slowly caudally through the DA and returning sporadically and haltingly through the caudal vein plexus. Blood cells do not transit from the DA to the DLAV along the ISVs, unlike control embryos. The reddish appearance of the tissue in the caudal vein plexus (white arrows) appears to be due to the accumulation of RBCs.
(7Z) Click here for additional data file.

Video S4 **Rescue by** ***meis1.1*** **mRNA of trunk blood flow in** ***hoxd4a*** **morphant embryos.** Video first showing vigorous circulation through the blood vessels of a control embryo at 48 hpf, followed by the absence of blood and weak circulation in a *hoxd4a* morphant. The last clip demonstrates significant rescue of blood cell count and vasculature in *hoxd4a* morphants rescued by co-injection of *meis1.1* mRNA, including the ability of blood cells to traverse from the DA to the DLAV along intact ISVs.
(7Z) Click here for additional data file.
