# Supplementary material for: Zebrafish hoxd4a Acts Upstream of meis1.1 to Direct Vasculogenesis, Angiogenesis and Hematopoiesis
Source: PLoS One. 2013 Mar 15;8(3):e58857. doi: 10.1371/journal.pone.0058857 (PMC3598951; doi:10.1371/journal.pone.0058857)
Supplement: File S1 — Supporting information. Contains supplementary Figures S1 to S7 and supplementary Tables S1 and S2. (PDF) [file pone.0058857.s001.pdf]

Fig S1

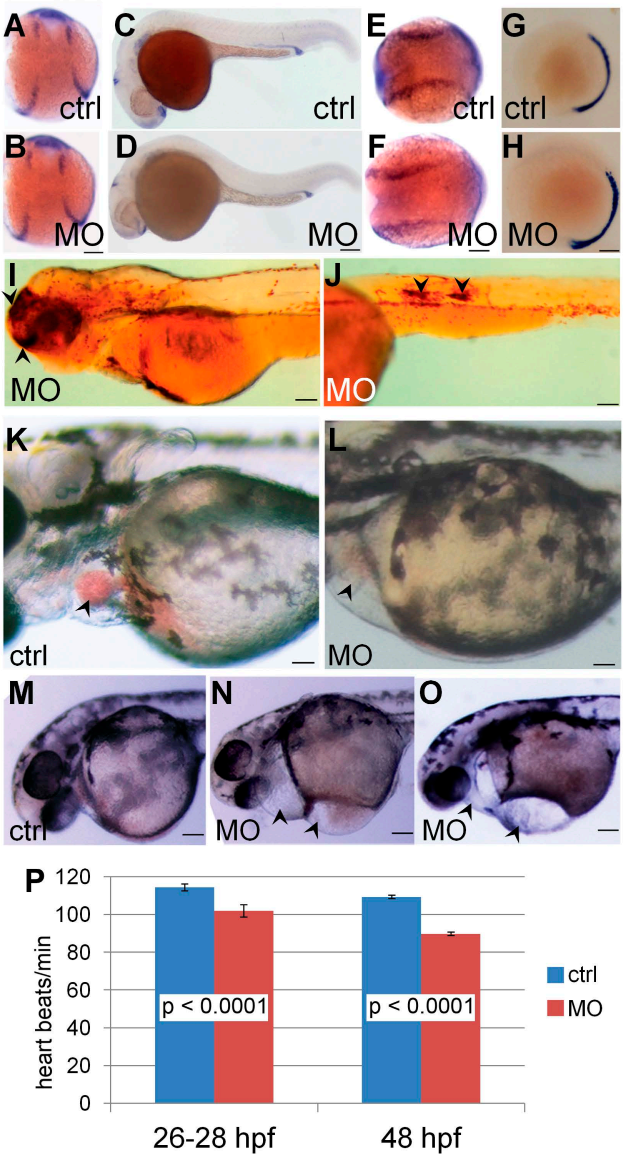
A
ctrl
B
MO
C
ctrl
D
MO
E
ctrl
F
MO
G
ctrl
H
MO
I
MO
J
MO
K
ctrl
L
MO
M
ctrl
N
MO
O
MO
P
heart beats/min
120
100
80
60
40
20
0
p < 0.0001
p < 0.0001
26-28 hpf
48 hpf
ctrl
MO

Fig S2

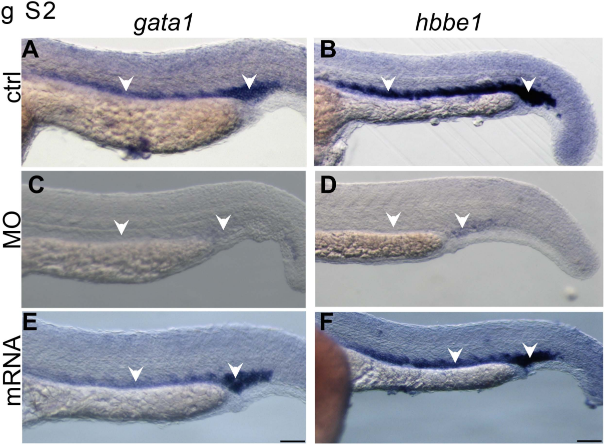

Fig S3

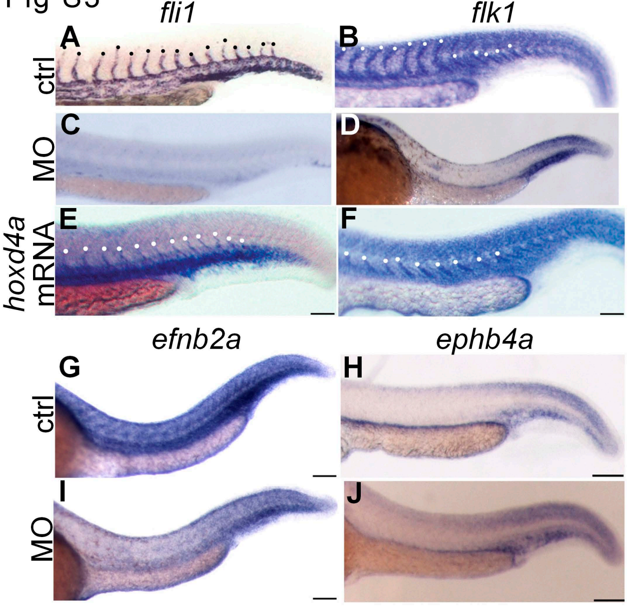
fli1
flk1
A
B
ctrl
C
D
MO
E
F
hoxd4a mRNA
efnb2a
ephb4a
G
H
ctrl
I
J
MO

Fig S4

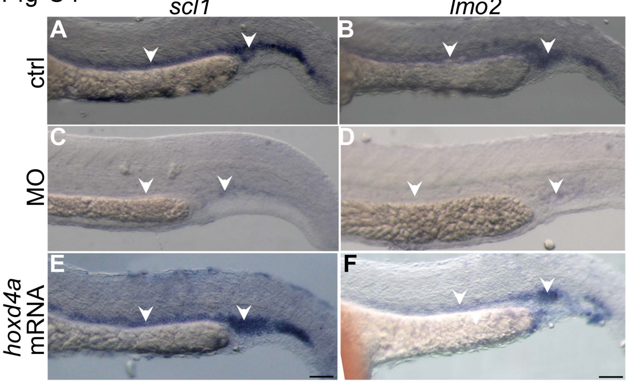

Fig S5

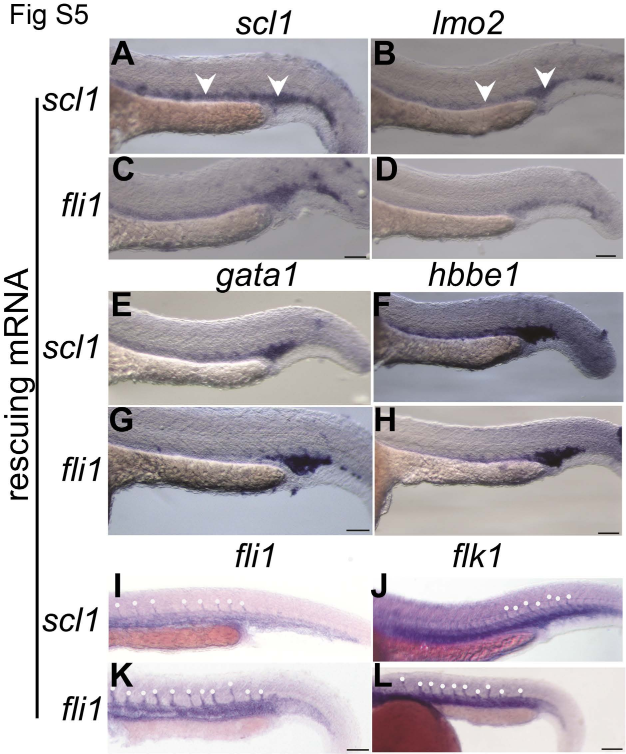

scl1
lmo2
gata1
hbbe1
fli1
flk1
A
B
C
D
E
F
G
H
I
J
K
L
rescuing mRNA
scl1
fli1
scl1
fli1
scl1
fli1

Fig S6

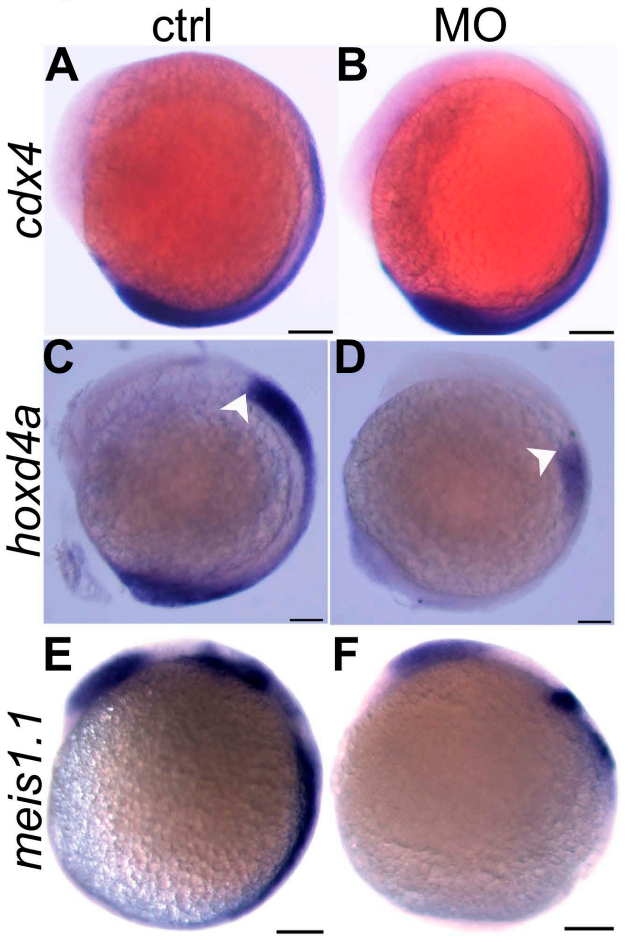

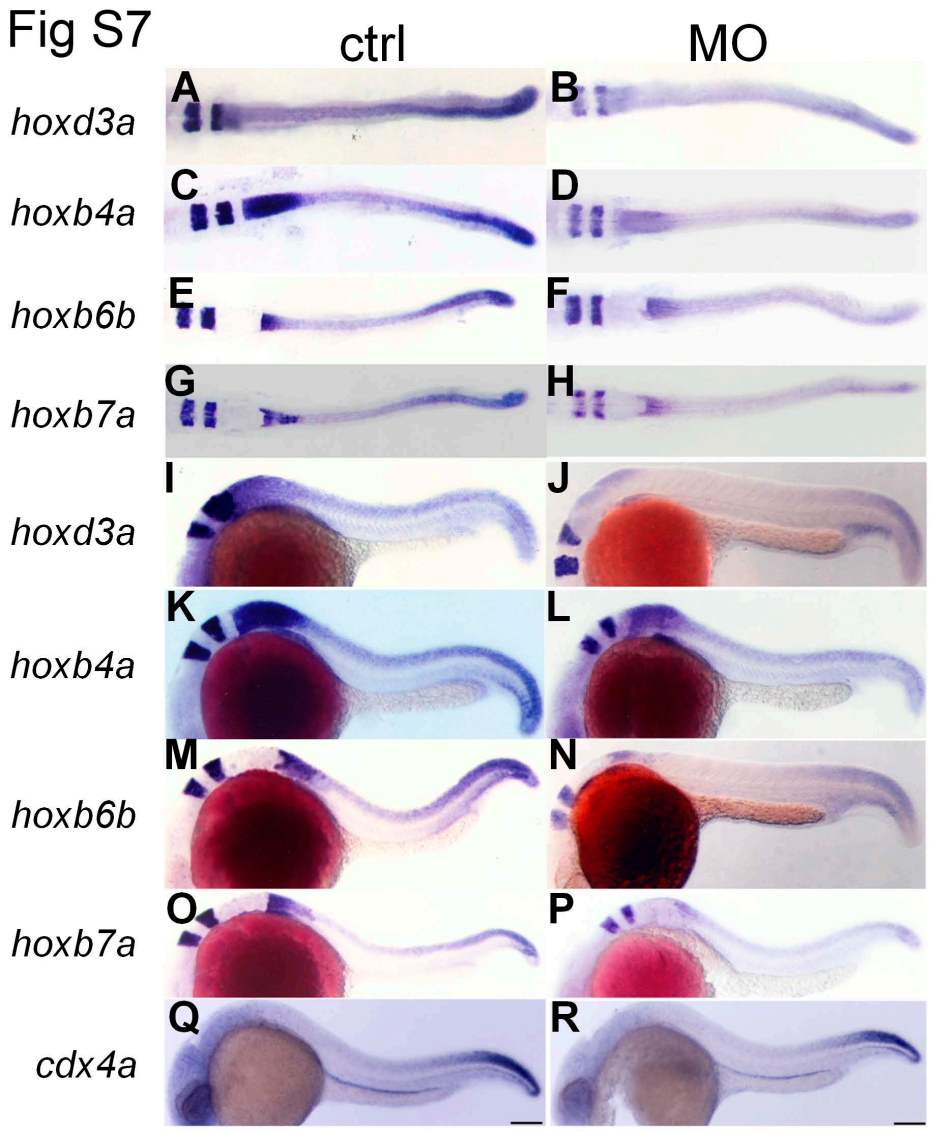
Fig S7
ctrl
MO
A
B
hoxd3a
C
D
hoxb4a
E
F
hoxb6b
G
H
hoxb7a
I
J
hoxd3a
K
L
hoxb4a
M
N
hoxb6b
O
P
hoxb7a
Q
R
cdx4a

Supplementary tables S1 and S2

Table S1. Primers used for cDNA cloning

Gene	Forward 5'→3'	Reverse 5'→3'
hoxd4a	CGCAGAATTCATGGCCATGAGT TCGTACATG	TTATAAAGTTGTGATCTCTGTTCTA GAGCGC
nkx2.5	CAT GGC ATC AGA GCT TGG TGA ACA AAA CTA CAT TTA CCG ACA GC	GAA TAA TAC GAC TCA CTA TAG GGA GCT CAC TTT CCG CAC AAA GGA AAA AAT AA

Table S2. Primers for quantitative RT-PCR

Gene	5' Forward 3'	5' Reverse 3'
β-actin	TTCCTTCCTGGGTATGGAATC	GCACTGTGTTGGCATACAGG
scl	CTATTAACCGTGGTTTTGCTGG	CCATCGTTGATTTCAACCTCAT
lmo2	GGACGCAGGCTTTACTACAAAC	CCGGATCCTCTTTTCACAGGAA
gata1	AAGATGGGACAGGCCACTAC	TGCTGACAATCAGCCTCTTTT
hbbe1	TGCTCTCTCCAGGATGTTGA	TCACAGTCTTGCCGTGTTTC
fli1	CAACGGATCCAGAGAGTCG	CCATGTAGCCAGTATAGTTCATCTG
flk1	TTCTGTCGGCAAGGAGTTTT	GACCGACCAGACATGAAACC
cmyb	CCGACAGAAGCCGGATGA	TGGCACTTCGCCTCAACTG
runx1	CGGTGAACGGTTAATATGAC	CTTTTCATCACGGTTTATGC
gata5	CGCTGATCAGGAAACCATC	GGGACGGGTTGGAAGATAAA
efnb2a	CCCATTTCCCCCAAAGACTA	CTTCCCCATGAGGAGATGC
vegf	TGCTCCTGCAAATTCACACAA	ATCTTGGCTTTTCACATCTGCAA
hoxd4a	ACCCCTAGCCCTTTCCCTG	GGTCTTTGTGTTTTGTTGTTGTCC
cdx4	CCACCACAGAACTTTGTTTCCA	AATCGGGCGAGCAATAGG
hoxb1a	ATCCCCCCAAAACAGGTAAA	CTCAAGTGTGGCAGCAATCT
hoxb3a	TTCACTGCTTCGCTCTAATGG	AGGGAGGGGGGTTGTCTTC
hoxd3a	TCGCGTCGTCTGTTACTCAC	AGAAATGCCTTCTGCTCCAA
hoxb4a	CTGCGGTCAGACTCCCACTA	GTTCGGGCTCACGATGTTTA
hoxc4a	TCCACTATAATCGCTACTTAACAC G	GGAGGACGAGGATCTGACTTTG
hoxb5a	ACATTTTACAGAAATCGACGAGG	CGCCGAGGTAGTGGAGGTT
hoxc5a	CGGGAGGCGATAAACACAA	GAGAGCCCAGAGCTGAACAAG
hoxb6b	AGGACAGGAGTCTTTCTTGGGTC	GAGCGGTCAGCGGTTCG
hoxc6a	TTATCTGGTGGGCAAGAGGT	GGTGAATAGAAAGGGGACGC
hoxb7a	CTTCATCATCTTCTGTCTCCCTG	TAGCCCCTCTGCTCTTCCTT
hoxb8a	ACCTACAGCCGCTATCAGACG	TCAGACTTGCTGCTTGGAAAC
hoxc8a	AAGACGGTGAAGCGGAGGA	TACATTTGTATGGAGGGGGATT
hoxa9a	CCCAACGCACTTCTCCACT	GTATTCCGTGCCGTCATCAT
hoxc9a	ACCATCCATACACTCACCAACC	CCCGTAGTGCCTGCTATTCC
hoxa10a	ACGGGGGGAAGTAAACCAC	TGACCTTTGCTCTCTTGTCGG
hoxd10a	ACGGGGGGAAGTAAACCAC	TGACCTTTGCTCTCTTGTCGG
meis1.1	GAG GAC ACA CAT CGC ACA GT	GAG CCA TGC CCT CAT AAT GT
