# Supplementary figures and images for: Zebrafish hoxd4a Acts Upstream of meis1.1 to Direct Vasculogenesis, Angiogenesis and Hematopoiesis
Source: PLoS One. 2013 Mar 15;8(3):e58857. doi: 10.1371/journal.pone.0058857 (PMC3598951; doi:10.1371/journal.pone.0058857)

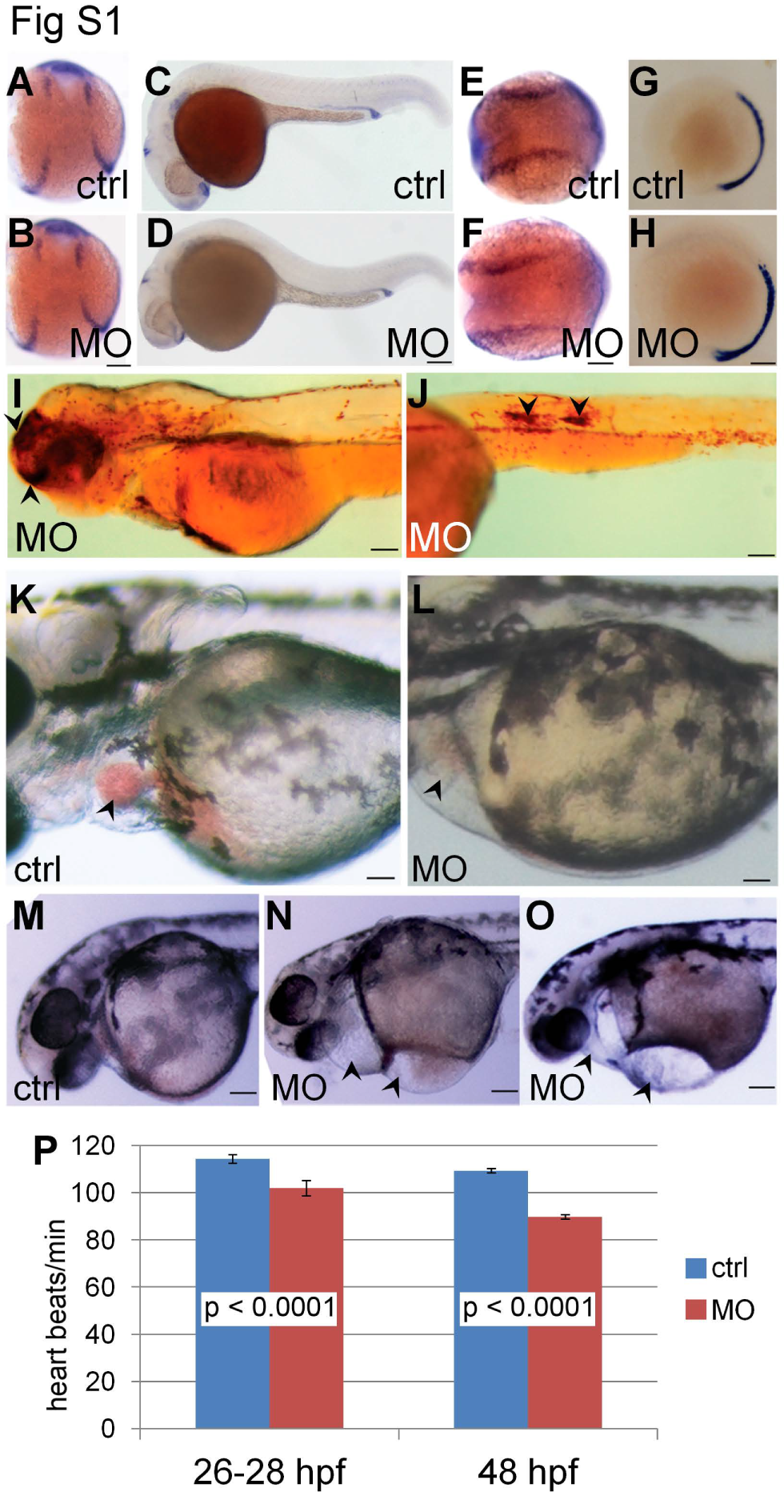

Supplement: Figure S1 — Patterning and morphology in hoxd4a morphants. (A–H) Knockdown of hoxd4a does not perturb overall patterning of the mesoderm. (A–D) Expression of pax2.1 shows that intermediate mesoderm forms and matures normally in hoxd4a morphants at 13 hpf (B) and 26–28 hpf (D) vs controls (A,C). (E,F) nkx2.5 expression in the precardiac lateral plate mesoderm at 13 hpf is normal in control (E) and morphant embryos (F). (G,H) myod expression in paraxial mesoderm is normal in control (G) and morphant embryos (H). Images in C, D, G and H are lateral views with anterior to the left. A, B, E and F show dorsal views with anterior to the top (A,B) or left (E,F). (I to O) Lateral views of control and hoxd4a-MO-injected larvae at 72 hpf. (I,J) Staining of hemoglobin with o-dianisidine reveals areas of hemorrhage such as in the head (I, arrowheads) and trunk (J, arrowheads) in some hoxd4a morphants. (K,L) Control larvae (K) but not hoxd4a morphants (L) show abundant RBCs passing through the heart (arrowheads). (M–O) Unlike control larvae (M), hoxd4a morphants display pericardial edema and edema over the adjacent yolk (N,O, arrowheads). Scale bars equal 100 µm. (P) The heart rate in morphants at 26–28 and 48 hpf was mildly reduced, but in a statistically significant manner as determined by unpaired Student’s t test (p<0.0001). Error bars give standard deviation. (TIF) [file pone.0058857.s002.tif]

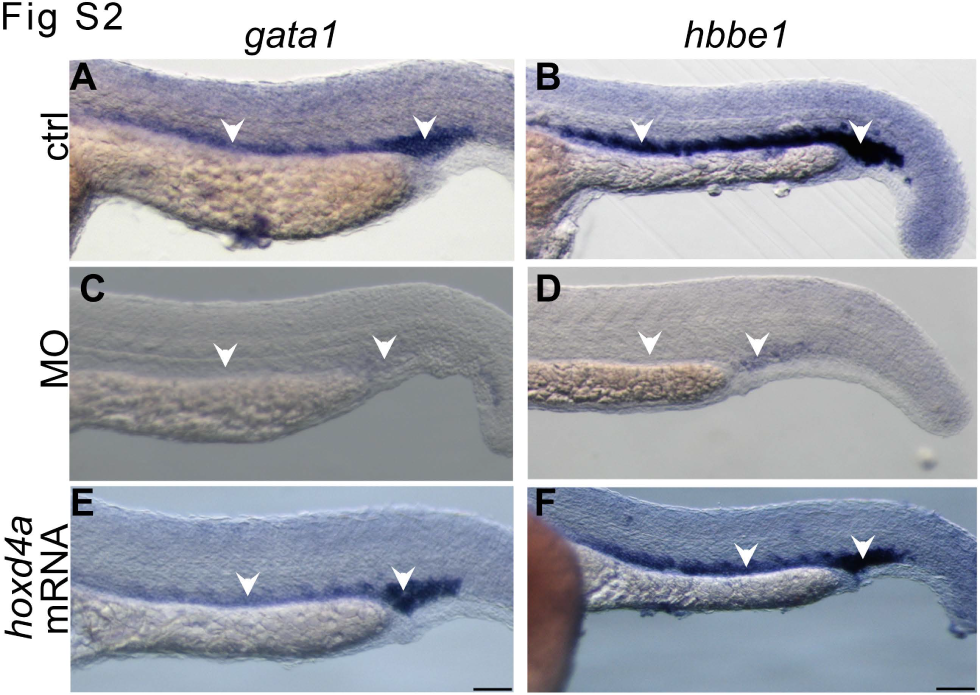

Supplement: Figure S2 — Reduced expression of markers of primitive hematopoiesis gata1 and β embryonic globin ( hbbe1 ) in hoxd4a morphants. WISH in control and morphant embryos at 26–28 hpf showing the expression of gata1 (A,C,E) and hbbe1 (B,D,F) in the ICM and PBI (white arrowheads). Normal expression of gata1 and hbbe1 (A,B) is severely reduced in hoxd4a morphants (C,D) and rescued by co-injection with capped mRNA for hoxd4a (E,F). All images are lateral views with anterior to the left. ctrl, embryos injected with a non-specific morpholino. MO, embryos injected with the anti-hoxd4a morpholino. hoxd4a mRNA, embryos simultaneously injected with the anti-hoxd4a MO plus capped mRNA for hoxd4a. Scale bars equal 100 µm. All images are at the same magnification. (TIF) [file pone.0058857.s003.tif]

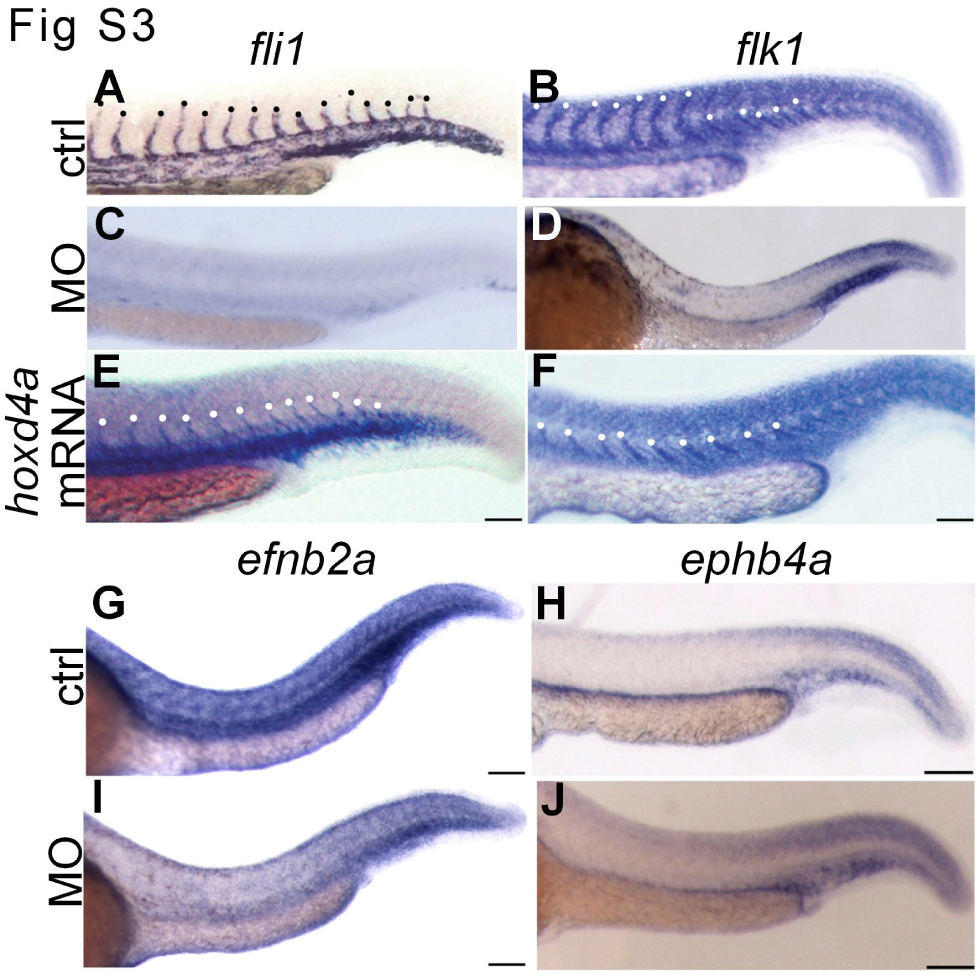

Supplement: Figure S3 — Reduced expression of markers of angiogenesis and venous specification in hoxd4a morphants. WISH in control and morphant embryos at 26–28 hpf showing the expression of fli1 (A,C,E) and flk1 (B,D,F). Normal expression of fli1 and flk1 (A,B) is severely reduced in hoxd4a morphants (C,D) and rescued by co-injection with capped mRNA for hoxd4a (E,F). White or black dots denote the tips of dorsally sprouting ISVs. Relative to controls (G,H), the expression of the arterial marker efnb2a is reduced in morphants at 26–28 hpf (I), while the venous marker ephb4a in morphants has recovered (J). Scale bars equal 100 µm. (TIF) [file pone.0058857.s004.tif]

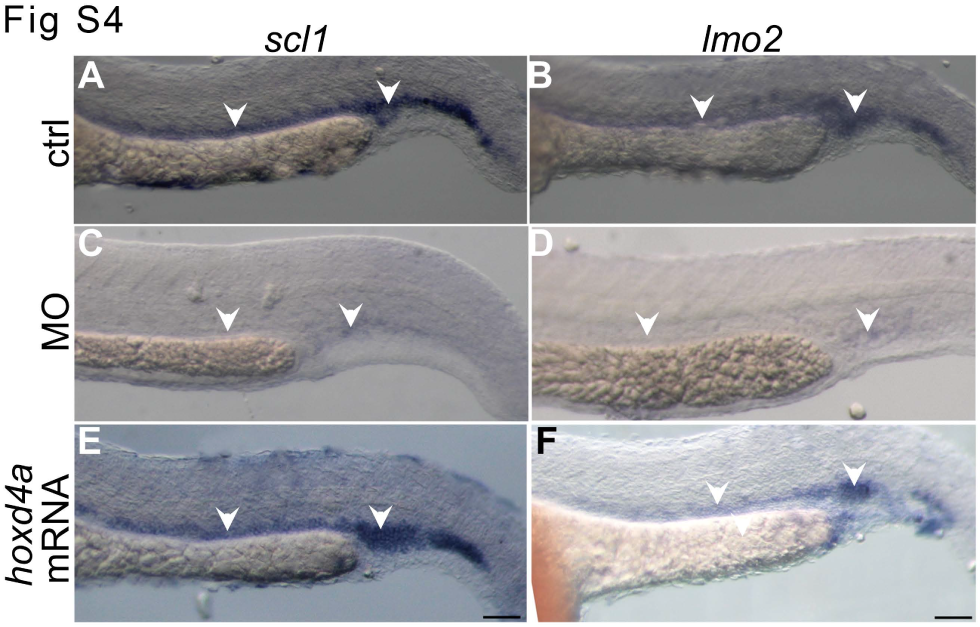

Supplement: Figure S4 — Reduced expression of scl and lmo2 in hoxd4a morphants at 26–28 hpf. (A–J) Expression analysis of scl (A,C,E) and lmo2 (B,D,F) at 26–28 hpf. Normal expression of scl and lmo2 (A,B) is severely reduced in hoxd4a morphants (C,D) and rescued by co-injection with capped mRNA for hoxd4a (E,F) All images present lateral views with anterior to the left and dorsal on top. Scale bars equal 100 µm. All images are at the same magnification. (TIF) [file pone.0058857.s005.tif]

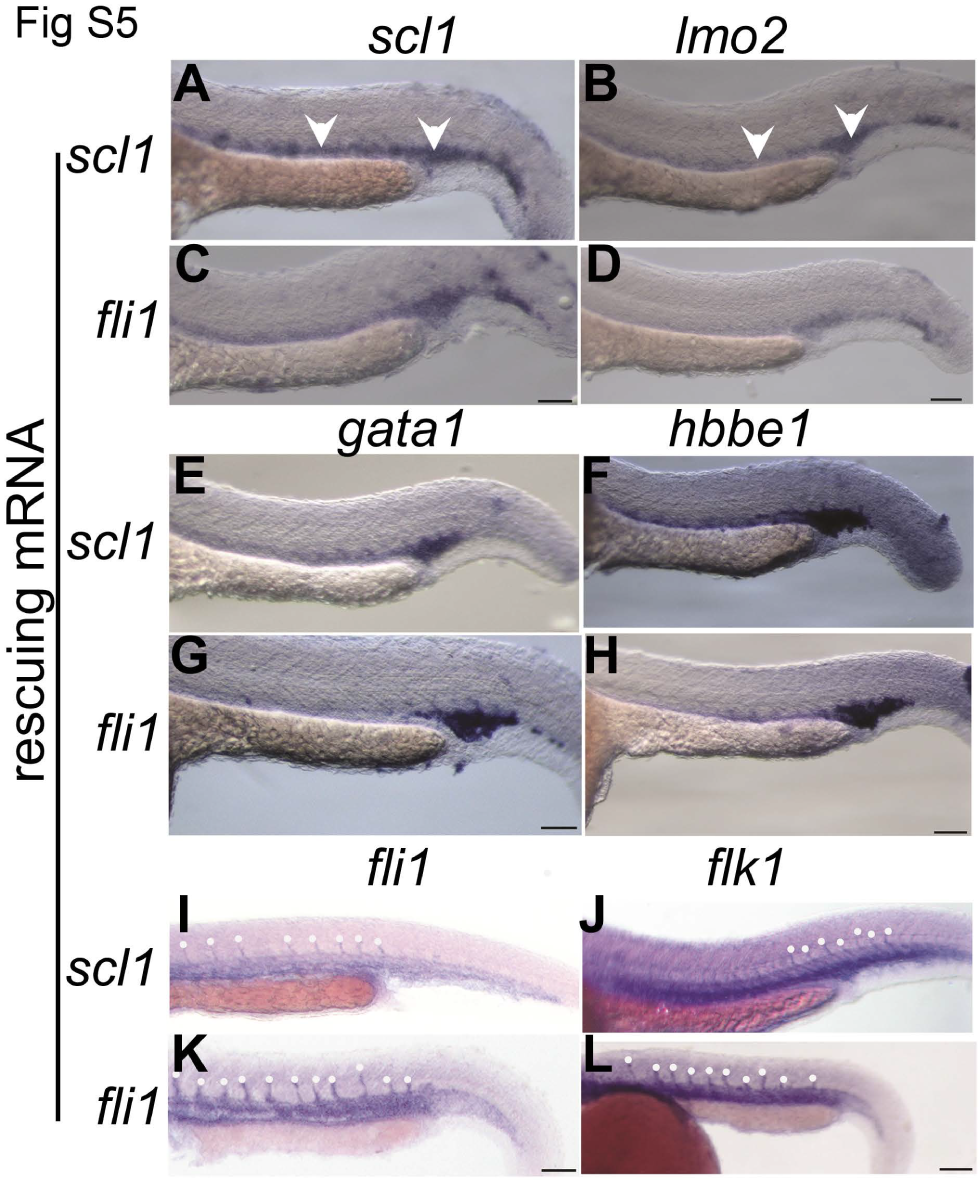

Supplement: Figure S5 — scl1 and fli1 act downstream of hoxd4a to direct formation of the hemangioblast. All images are of hoxd4a morphants at 26–28 hpf previously injected with capped mRNAs for either scl1 or fli1 as indicated on the left. WISH was performed to detect expression of scl1 and lmo2 (A–D), gata1 and hbbe1 (E–H) and fli1 and flk1 (I–L). Scale bars equal 100 µm. All images are at the same magnification. (TIF) [file pone.0058857.s006.tif]

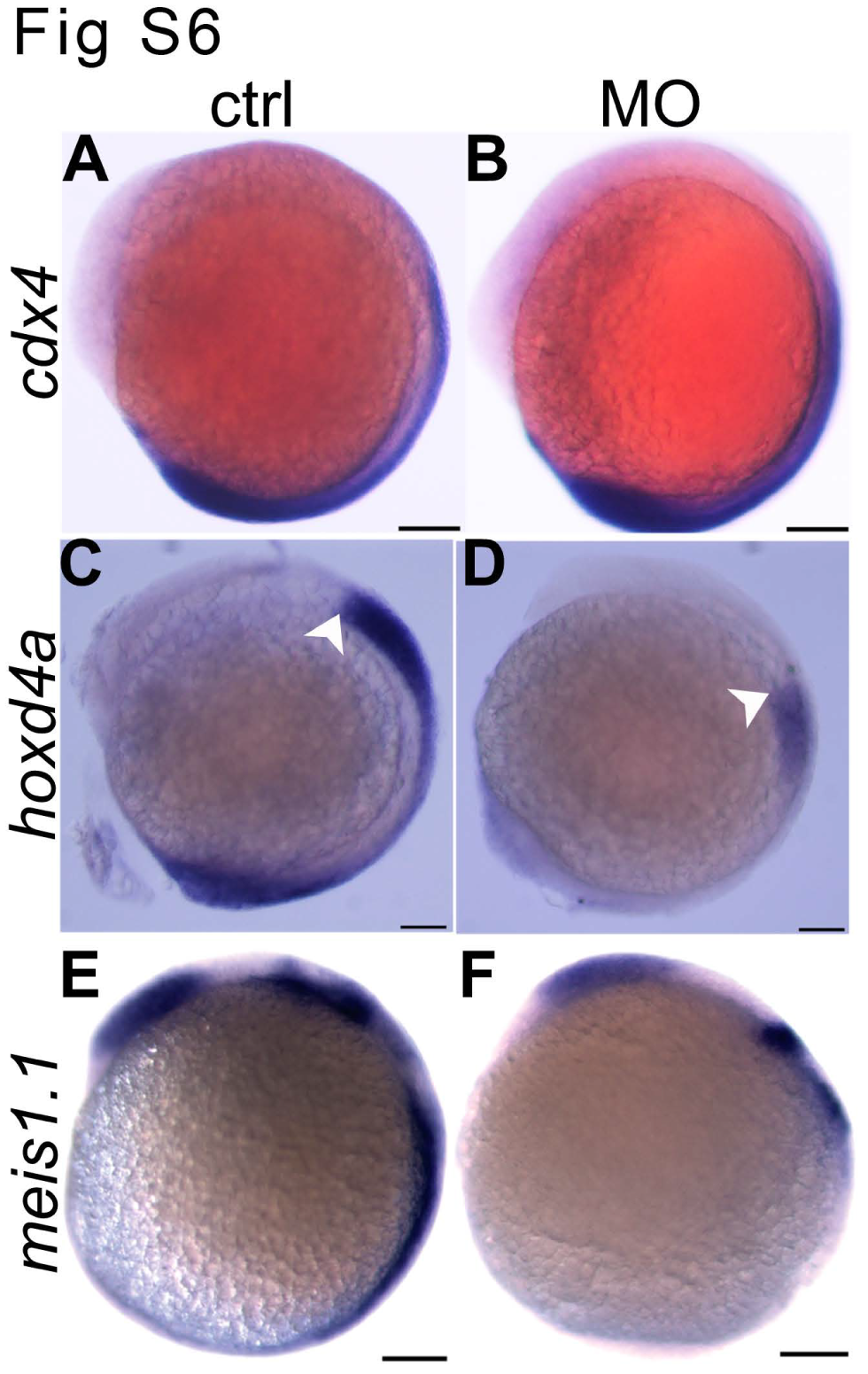

Supplement: Figure S6 — Knockdown of hoxd4a results in decreased expression of meis1.1 but not cdx4 at 13 hpf (∼8 somites). (A–F) Expression of cdx4 (A,B), hoxd4a (C,D) and meis1.1 (E,F) in control (A,C,E) and hoxd4a morphants (B,D,F) at the shield stage. The white arrowheads in C and D denote the hoxd4a anterior expression boundary in the hindbrain. Scale bars equal 100 µm. All images are at the same magnification. (TIF) [file pone.0058857.s007.tif]

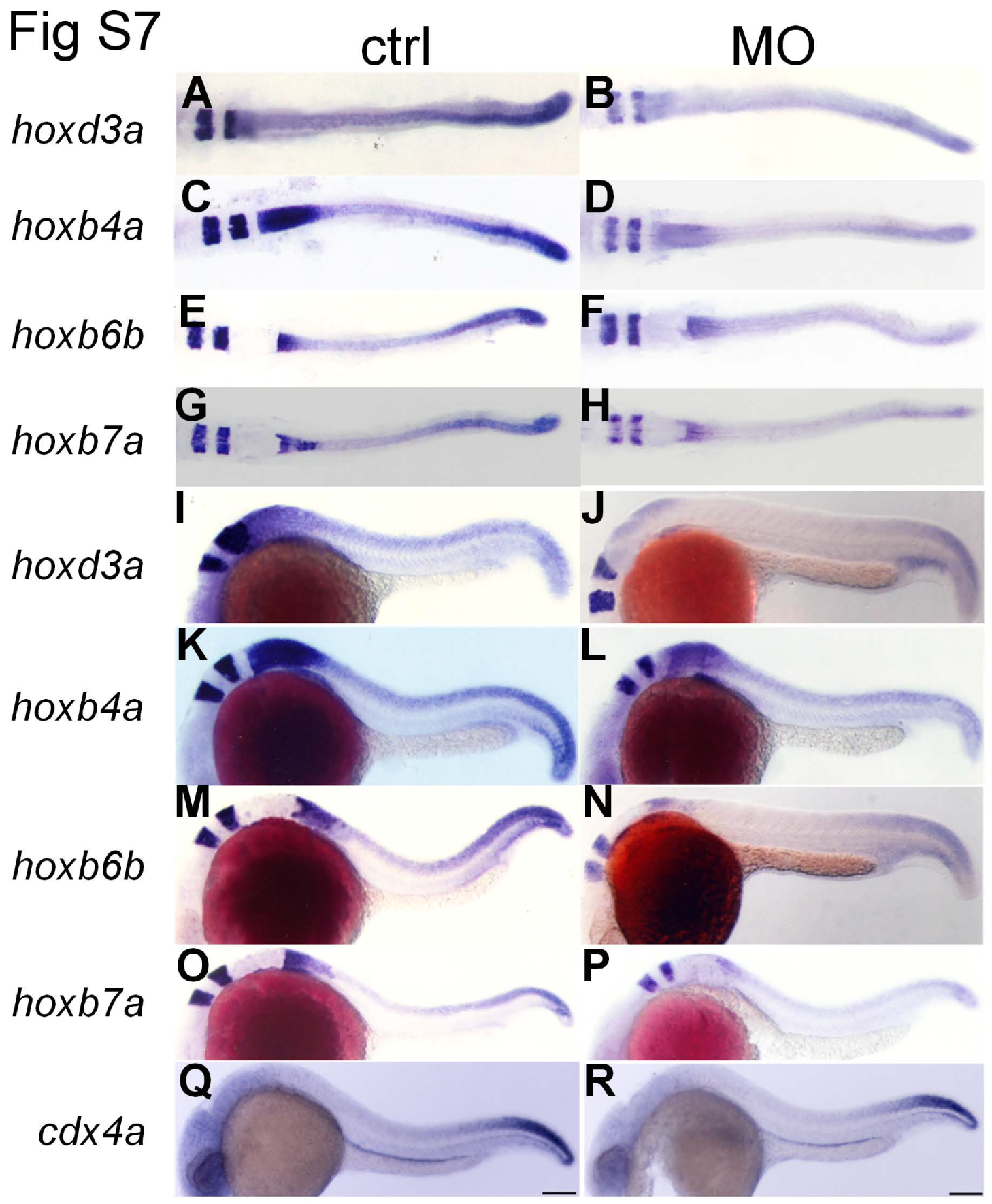

Supplement: Figure S7 — The expression of multiple hox genes is reduced at 26–28 hpf in hoxd4a morphants. Images are dorsal views (A–H) and lateral views (I–P) of embryos taken through in situ hybridization for the indicated hox genes. Relative to control embryos (A,C,E,G,I,K,M,O), hox gene expression is reduced in hoxd4a morphants (B,D,F,H,J,L,N,P). All embryos were simultaneously probed for krox20a expression in r3 and r5 as in Figure 1C. (Q–R) cdx4 expression is unchanged in control (Q) and hoxd4a morphants (R) at 26–28 hpf. Scale bars equal 100 µm. All images are at the same magnification. (TIF) [file pone.0058857.s008.tif]

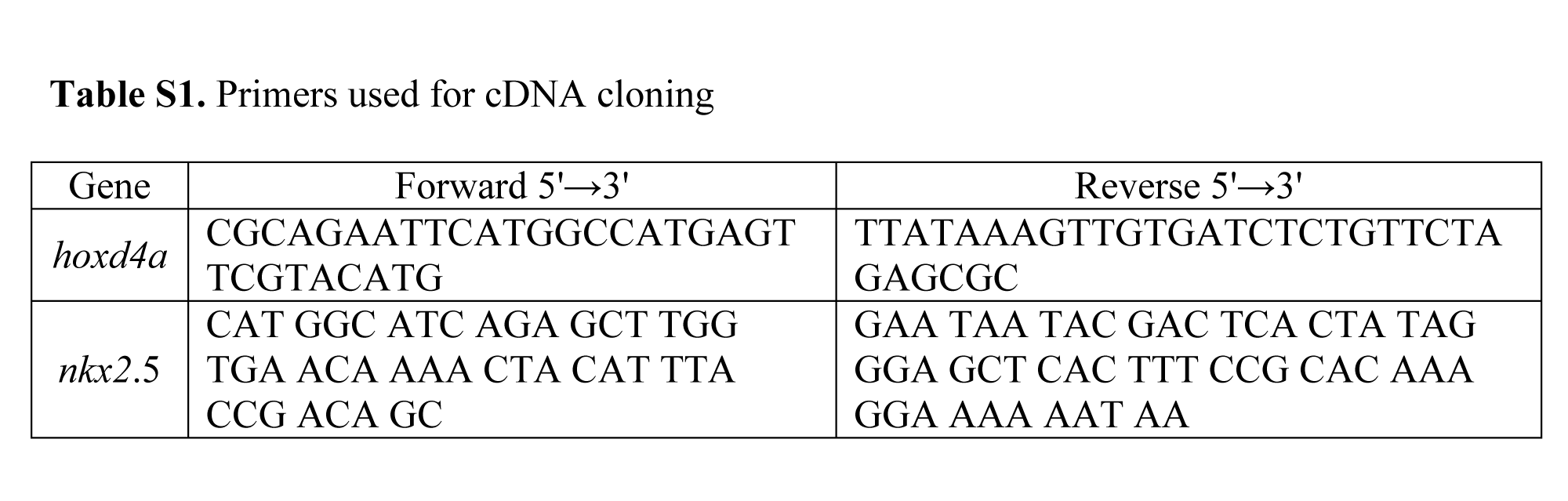

Supplement: Table S1 — Primers used for cDNA cloning. (TIF) [file pone.0058857.s009.tif]

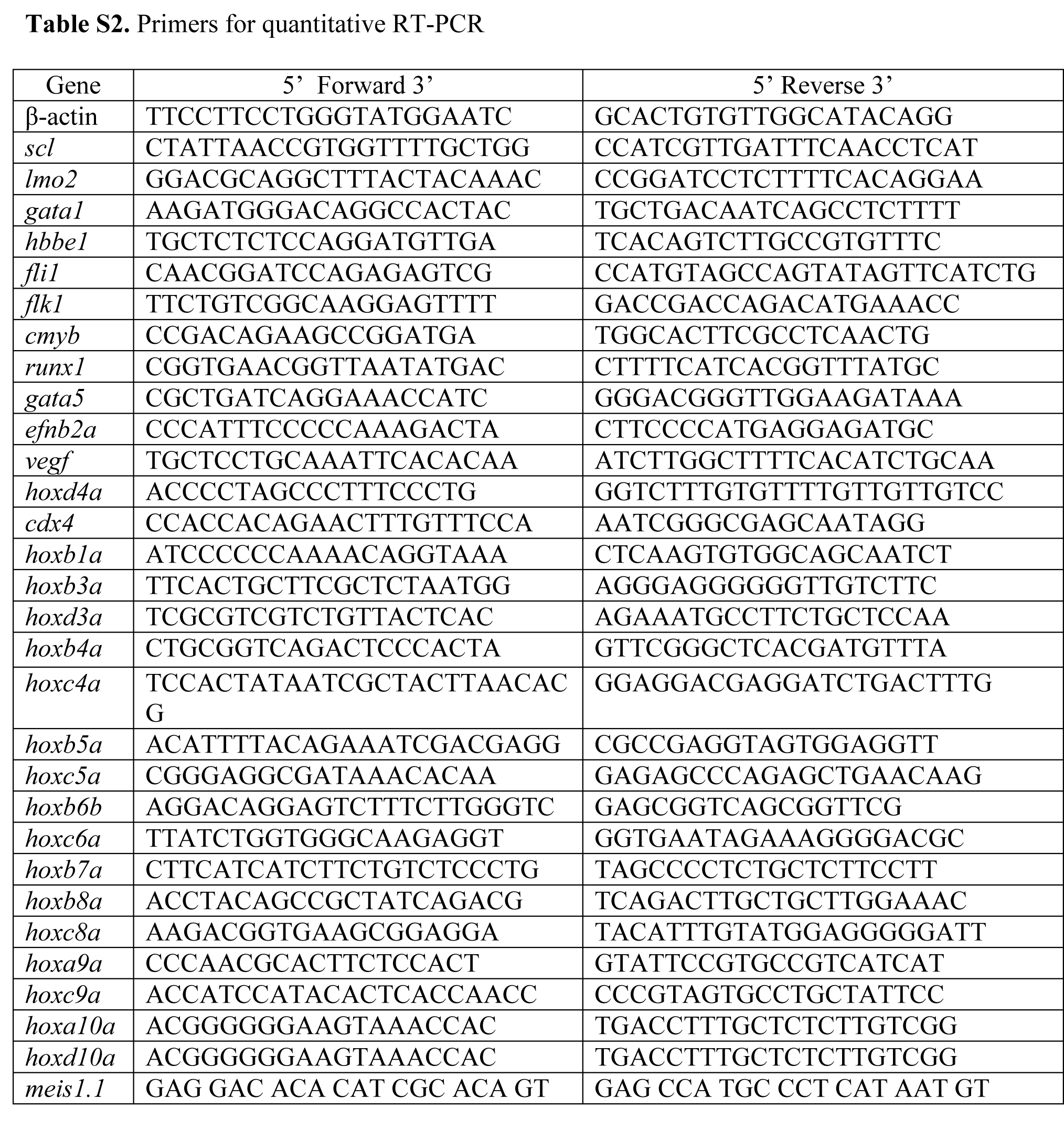

Supplement: Table S2 — Primers for quantitative RT-PCR. (TIF) [file pone.0058857.s010.tif]
